# Effect of the gut microbiome, plasma metabolome, peripheral cells, and inflammatory cytokines on obesity: a bidirectional two-sample Mendelian randomization study and mediation analysis

**DOI:** 10.3389/fimmu.2024.1348347

**Published:** 2024-03-15

**Authors:** Ying Li, Xin Wang, Zitong Zhang, Lei Shi, Liang Cheng, Xue Zhang

**Affiliations:** ^1^ Human Molecular Genetics Group, National Health Commission (NHC) Key Laboratory of Molecular Probes and Targeted Diagnosis and Therapy, The Fourth Affiliated Hospital, Harbin Medical University, Harbin, China; ^2^ Department of Child and Adolescent Health, School of Public Health, Harbin Medical University, Harbin, China; ^3^ National Health Commission (NHC) Key Laboratory of Molecular Probes and Targeted Diagnosis and Therapy, Harbin Medical University, Harbin, China; ^4^ College of Bioinformatics Science and Technology, Harbin Medical University, Harbin, China; ^5^ Department of Medical Genetics, College of Basic Medical Sciences, Harbin Medical University, Harbin, China

**Keywords:** obesity, gut microbiota, plasma metabolites, peripheral cells, inflammatory cytokines, Mendelian randomization, mediation analysis

## Abstract

**Background:**

Obesity is a metabolic and chronic inflammatory disease involving genetic and environmental factors. This study aimed to investigate the causal relationship among gut microbiota abundance, plasma metabolomics, peripheral cell (blood and immune cell) counts, inflammatory cytokines, and obesity.

**Methods:**

Summary statistics of 191 gut microbiota traits (N = 18,340), 1,400 plasma metabolite traits (N = 8,299), 128 peripheral cell counts (blood cells, N = 408,112; immune cells, N = 3,757), 41 inflammatory cytokine traits (N = 8,293), and 6 obesity traits were obtained from publicly available genome-wide association studies. Two-sample Mendelian randomization (MR) analysis was applied to infer the causal links using inverse variance-weighted, maximum likelihood, MR-Egger, weighted median, weighted mode, and Wald ratio methods. Several sensitivity analyses were also utilized to ensure reliable MR results. Finally, we used mediation analysis to identify the pathway from gut microbiota to obesity mediated by plasma metabolites, peripheral cells, and inflammatory cytokines.

**Results:**

MR revealed a causal effect of 44 gut microbiota taxa, 281 plasma metabolites, 27 peripheral cells, and 8 inflammatory cytokines on obesity. Among them, five shared causal gut microbiota taxa belonged to the phylum *Actinobacteria*, order *Bifidobacteriales*, family *Bifidobacteriaceae*, genus *Lachnospiraceae* UCG008, and species *Eubacterium nodatum* group. Furthermore, we screened 42 shared causal metabolites, 7 shared causal peripheral cells, and 1 shared causal inflammatory cytokine. Based on known causal metabolites, we observed that the metabolic pathways of D-arginine, D-ornithine, linoleic acid, and glycerophospholipid metabolism were closely related to obesity. Finally, mediation analysis revealed 20 mediation relationships, including the causal pathway from gut microbiota to obesity, mediated by 17 metabolites, 2 peripheral cells, and 1 inflammatory cytokine. Sensitivity analysis represented no heterogeneity or pleiotropy in this study.

**Conclusion:**

Our findings support a causal relationship among gut microbiota, plasma metabolites, peripheral cells, inflammatory cytokines, and obesity. These biomarkers provide new insights into the mechanisms underlying obesity and contribute to its prevention, diagnosis, and treatment.

## 1 Introduction

Obesity, a complex metabolic disease, arises from an imbalance between energy intake and expenditure, leading to excess energy storage in adipose tissues. Its etiology is multifaceted, involving both genetic and environmental factors. Presently, approximately one-third of the global population is overweight (defined as a body mass index [BMI] between 25 and 29 kg/m²), with 10% classified as obese (BMI ≥ 30 kg/m^2^) (1). This global epidemic poses significant risks to physical and mental health, being a primary contributor to various diseases, including cardiovascular issues, allergic conditions, hypertension, type 2 diabetes (T2D), cancer, and mood-related disorders (2–4). Thus, obesity is a serious public health concern.

In recent years, increasing evidence has shown that an imbalance in the gut microbiota may play a major role in obesity (5). The gut microbiota is a microbial community living in the human intestine that plays an important role in human metabolic regulation and immunomodulation via interactions with the host (6, 7). The diversity and richness of the gut microbiota in obese patients are reduced, and the composition of the gut microbiota changes to varying degrees (8, 9). For example, an increased *Firmicutes* to *Bacteroidetes* ratio may play a role in the development of obesity (8, 10). A case-control study found that *Enterobacteriaceae* levels were increased, whereas *Desulfovibrio* and *Akkermansia muciniphila* levels were decreased in overweight and obese children (11).

Metabolomics can reveal correlations between metabolites or metabolic pathways and physiological and pathological changes, thus providing new information for research on disease mechanisms (12). Multiple studies have shown that metabolites and metabolic pathways are closely associated with obesity and that obese patients have metabolic disorders (13, 14). For example, a study using targeted serum metabolomics identified metabolites significantly associated with obesity. In that study, serum concentrations of glycine, glutamine, and glycero-phosphatidylcholine (Pcaa) 42:0 were positively correlated, whereas those of PCaa 32:0, PCaa 32:1, and PCaa 40:5 were negatively correlated with obesity (14). In addition, plasma metabolites, such as branched-chain amino acids and glutamate, may mediate the relationship between the gut microbiota and obesity (15).

Obesity is a chronic inflammatory disease closely related to the immune system and inflammatory responses (16). Adipose tissue macrophages are key contributors to obesity-related inflammation, accounting for less than 10% of the immune cells in lean individuals and up to 50% in obese individuals (17). Additionally, a higher white blood cell count may be associated with an increased risk of obesity. After weight loss, total white blood cells, major components, neutrophils, and lymphocytes significantly decrease (18, 19). There is also an increasing number of reports on the relationship between inflammatory cytokines and the risk of obesity. Previous studies have shown that the increase in the levels of pro-inflammatory cytokines interleukin (IL)-1, IL-6, and tumor necrosis factor alpha is closely related to the occurrence and development of obesity (20, 21). In addition, research has shown decreased serum levels of IL-27 in obese individuals. IL-27 can act directly on adipocytes and lead to adipocyte differentiation and thermogenesis, thus reducing weight and improving metabolic diseases, such as T2D (22).

While previous studies have identified associations between the gut microbiome, metabolome, immune inflammation, and obesity, the precise causal relationships and their respective mediation proportions remain unclear. Mendelian randomization (MR) analysis is an effective method that uses genetic variation as an instrumental variable (IV) to evaluate the potential causal relationship between exposures and outcomes (23). This minimizes the impact of confounding factors on causal estimation, as genetic variations are randomly assigned at conception. Mediation analysis is used to evaluate the effects of an exposure on an outcome through a mediator (24). Therefore, we conducted MR analyses based on publicly available genome-wide association study (GWAS) summary data to evaluate the causal relationship among the gut microbiota, plasma metabolites, peripheral cells, inflammatory cytokines, and obesity, and to identify pathways from the gut microbiota to obesity mediated by plasma metabolites, peripheral cells, and inflammatory cytokines.

## 2 Methods

### 2.1 Study design

**Figure 1:**
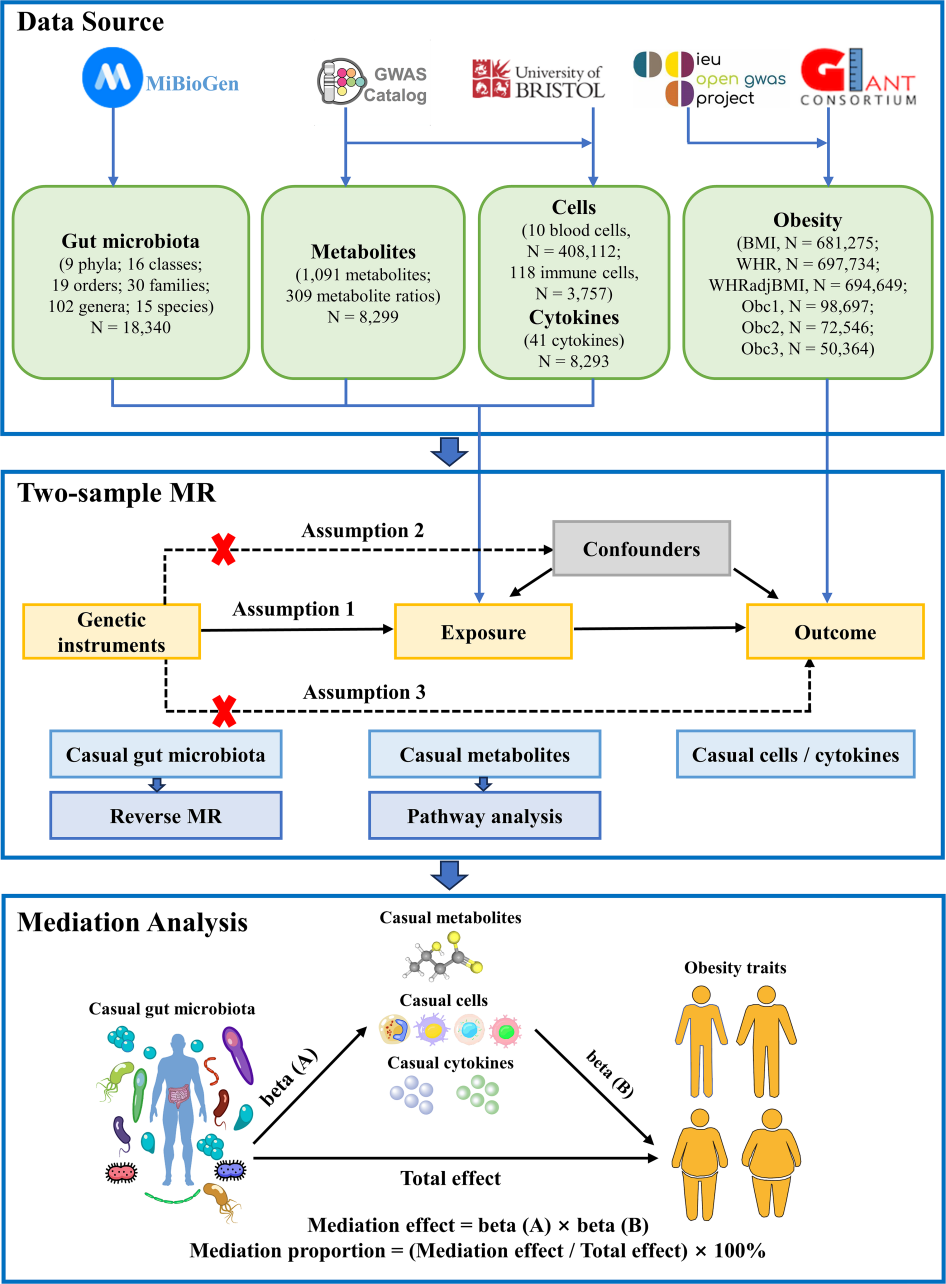
Flow chart of the study. Mendelian randomization study rationale: assumption 1, genetic instruments are associated with exposure; assumption 2, genetic instruments are not associated with confounders; assumption 3, genetic instruments are not associated with outcome, and genetic instruments act on outcome only through exposure. BMI, body mass index; WHR, waist-to-hip ratio; WHRadjBMI, WHR adjusted for BMI; Obc1, Obesity class 1; Obc2, Obesity class 2; obc3, Obesity class 3; MR, Mendelian randomization.

The study flowchart is illustrated in **Figure 1**. First, we obtained published GWAS summary data that included traits such as gut microbiota, plasma metabolites, peripheral cells, inflammatory cytokines, and obesity (**Supplementary Table S1**). Second, two-sample MR analyses were used to evaluate the causal relationship among gut microbiota, plasma metabolites, peripheral cells, inflammatory cytokines, and obesity. Finally, two-step and multivariable MR (MVMR) analyses were used to identify the mediation effect of plasma metabolites, peripheral cells, and inflammatory cytokines on the relationship between gut microbiota and obesity. Our MR study was conducted in accordance with the STROBE-MR guidelines (**Supplementary Table S2**) (25).

### 2.2 Data sources

The summary statistics of gut microbiota were retrieved from the largest multi-ethnic GWAS meta-analysis exploring the host genetic impact on gut microbiota, which was based on the MiBioGen consortium (https://mibiogen.gcc.rug.nl/), including 18,340 individuals from 24 cohorts (26). The gut microbiota was identified using 16S rRNA sequencing, and the patients were genotyped using a genome-wide single nucleotide polymorphism (SNP) microarray to determine the genetic locus affecting the relative abundance of the gut microbiota. The GWAS summary data of 191 gut microbiome components (including 9 phyla, 16 classes, 19 orders, 30 families, 102 genera, and 15 species) were included in this study for subsequent MR analyses.

Summary statistics of plasma metabolomics were acquired on the GWAS Catalog (https://www.ebi.ac.uk/gwas/) under the study accession numbers GCST90199621–GCST90201020, which included 1,091 plasma metabolites and 309 metabolite ratios from 8,299 European individuals (27). In that study, there were 850 known metabolites among 1,091 plasma metabolites, which could be divided into 8 broad metabolic groups: lipid (395), amino acid (210), xenobiotics (130), nucleotides (33), cofactors and vitamins (31), carbohydrates (22), peptides (21), and energy (8); the remaining metabolites were partially characterized molecules (21) and unknown (220).

Summary statistics for blood cell traits included 408,112 European participants (28); summary statistics for peripheral immune cells included that of 3,757 European individuals analyzed using flow cytometry (29). The GWAS data were downloaded from the GWAS Catalog, and we selected 10 blood cell count traits and 118 immune cell absolute count traits for subsequent analyses (accession numbers for each trait can be found in **Supplementary Table S1**). GWAS data for 41 inflammatory cytokines were collected from the University of Bristol (https://data.bris.ac.uk/data/dataset), including three Finnish cohort studies (N = 8,293): the Cardiovascular Risk in Young Finns Study, FINRISK1997, and FINRISK2002 (30, 31).

GWAS summary data for obesity-related traits were collected from large-scale GWAS or the corresponding meta-analyses. Obesity-related traits included BMI, waist-to-hip ratio (WHR), WHR adjusted for BMI (WHRadjBMI), and Obesity classes 1, 2, and 3. GWAS summary data on BMI (32), WHR (33), and WHRadjBMI (33) were obtained from the meta-analysis of UK Biobank and Genetic Investigation of Anthropometric Traits (GIANT) consortium (https://portals.broadinstitute.org/collaboration/giant/index.php/GIANT_consortium_data_files), which contained approximately 700,000 European individuals. Three obesity clinical classification datasets were downloaded at the IEU OpenGWAS database (https://gwas.mrcieu.ac.uk/) and obtained from a genome-wide meta-analysis (34), which contained 263,407 European individuals: Obesity class 1 (BMI ≥ 30 kg/m^2^) contained 32,858 patients and 65,839 controls; Obesity class 2 (BMI ≥ 35 kg/m^2^) included 9,889 patients and 62,657 controls; and Obesity class 3 (BMI ≥ 40 kg/m^2^) included 2,896 patients and 47,468 controls. Control was defined as an individual with a BMI < 25 kg/m^2^.

### 2.3 Instrumental variable selection

To estimate causal effects using genetic variation, three basic assumptions of IVs must be satisfied: 1) IVs are related to exposure factors; 2) IVs are not associated with confounding factors; and 3) IVs are not related to outcome variables and only act on outcome variables through exposure factors. Specifically, the IVs included in this study were screened to meet the following conditions: 1) The SNP obtained at the locus-wide significance threshold of *P* < 1 × 10^-5^ is used when there are too few whole-genome significance loci in the original GWAS results (35), or the genome-wide significance threshold of *P* < 5 × 10^-8^ is used as a potential tool variable related to each exposure trait. 2) SNPs related to outcome variables were excluded (*P* < 0.05). 3) The clumping process was performed to avoid the impact of linkage disequilibrium (r^2^ < 0.01, window size = 500 kb; or r^2^ < 0.001, window size = 10,000 kb). 4) The MR pleiotropy residual sum and outlier (MR-PRESSO) test was applied to detect horizontal pleiotropy, and the pleiotropy effect was eliminated by removing the outliers (36). In summary, the SNPs were sorted in ascending order according to the *P*-values of the MR-PRESSO outlier test, and the remaining were eliminated one by one until there was no pleiotropy (MR-PRESSO global test *P*-value > 0.05). 5) The strength of the selected SNPs was evaluated using F-statistic, where SNPs with F-statistic < 10 were excluded to avoid weak instrument bias in the MR analysis (37). The F statistic formula is F = [R^2^ × (n − k − 1)] / [k × (1 − R^2^)], where R^2^ is the portion of the exposure variance explained by the IVs, n is the sample size, and k represents the number of IVs (37). 6) IVs with a stronger association with the outcome than exposure were removed by Steiger filtering.

### 2.4 Statistical analysis

#### 2.4.1 Two-sample Mendelian randomization

The MR method was used to evaluate the causal relationship among the gut microbiota, plasma metabolites, peripheral cells, inflammatory cytokines, and obesity. The Wald ratio was used to infer the causality for exposure, which included only one IV. For exposure comprising multiple IVs, inverse variance-weighted (IVW), maximum likelihood, MR-Egger, weighted median, and weighted mode methods were used to infer causality. IVW usually provides the highest statistical power (38); therefore, it is preferred, whereas other methods are used as supplements. IVW uses a meta-analysis to combine the Wald ratio estimates of each IV, where the intercept is limited to zero (38). In the absence of horizontal pleiotropy, IVW can provide an unbiased causal estimate (39). When there was heterogeneity, the random-effect IVW test provided more conservative and robust estimates; otherwise, a fixed-effect model was used. Similar to IVW, the maximum likelihood method assumes a linear relationship between exposure and outcome (40). MR-Egger verifies the existence of multiple horizontal effects; when pleiotropy exists, it can provide an effective causal estimation (41). Even when up to 50% of the IVs are ineffective, the weighted median can provide effective causal estimates (42). The weighted mode approach is still valid if most IVs with similar causal estimates are valid instruments, even if other IVs do not meet the requirements of the MR analysis (43).

Sensitivity analysis was performed to assess the robustness of causality. MR-Egger regression and MR-PRESSO were used to assess the horizontal pleiotropy. The non-zero intercept of the MR-Egger regression suggested directional pleiotropy (41). Cochran’s Q test was used to assess the heterogeneity among the IVs. Additionally, leave-one-out sensitivity analysis was used to assess whether a single SNP drove the causal estimation. MR Steiger analysis was used to assess the direction of the potential causal association between exposure and outcomes. Only causal microbial characteristics, plasma metabolites, peripheral cells, and inflammatory cytokines with no heterogeneity or pleiotropy were included in the subsequent analysis when the IVW MR method results reached a significance threshold of *P* < 0.05.

Furthermore, considering the potential chance to increase the overall type I error during multiple comparisons, we implemented the false discovery rate (FDR) correction using the Benjamini–Hochberg procedure (44) on the primary IVW results. A significance threshold of FDR < 0.1 indicates a significant association, whereas *P*_IVW_ < 0.05 but FDR > 0.1 implies a suggestive association.

All MR analyses were performed in R (version 4.3.1) software, using the “TwoSampleMR” (version 0.5.7) (https://github.com/MRCIEU/TwoSampleMR) (39) and “MR-PRESSO” (version 1.0) (https://github.com/rondolab/MR-PRESSO) (36) packages.

#### 2.4.2 Reverse Mendelian randomization analysis

To explore whether obesity had a causal effect on the identified gut microbiota (*P*_IVW_ < 0.05), a reverse MR analysis was performed. In this scenario, obesity-related SNPs were regarded as IVs, obesity as the exposure, and gut microbiota taxa as the outcomes. The reverse MR analysis procedure was similar to that used for the MR analysis.

#### 2.4.3 Metabolic pathway analysis

For identified known plasma metabolites (*P*_IVW_ < 0.05), we used MetaboAnalyst 5.0 (https://www.metaboanalyst.ca/) (45) to conduct metabolic pathway analysis to identify potential metabolic pathways that may be related to the biological processes of obesity. This study used two libraries: the Small Molecule Pathway Database (SMPDB) (46) and the Kyoto Encyclopedia of Genes and Genomes (KEGG) database (47).

#### 2.4.4 Mediation analysis

Mediation analysis aims to evaluate the pathway from exposure to outcome through a mediator, which helps explore the potential mechanisms by which exposure affects outcome (24). The mediation analysis in this study focused on obesity-related gut microbiota, plasma metabolites, peripheral cells, and inflammatory cytokines. First, the causal relationship between gut microbiota and plasma metabolites, peripheral cells, and inflammatory cytokines was evaluated using two-sample MR methods to obtain beta (A). Second, MVMR was used to screen plasma metabolites, peripheral cells, and inflammatory cytokines that still had a causal relationship with obesity after correction for gut microbiota to obtain beta (B) and ensure that the mediating effects on outcomes are independent of exposure (24). The mediation effect was calculated using a two-step MR: mediation effect = beta (A) × beta (B). The total effect of the gut microbiota on obesity was obtained in the previous two-sample MR, and direct effect = (total effect − mediation effect). The mediation proportion used the following formula: mediation proportion = (mediation effect / total effect) × 100%. The 95% confidence intervals (CI) for the mediation effects and proportions mediated were estimated using the delta method (24). Based on the results, we categorized the identified mediators into different levels of evidence. When only a triangular relationship existed, representing that exposure was causally associated with outcome, mediator was causally associated with outcome, and exposure was causally associated with mediator. The identified metabolites, peripheral cells, or cytokines were considered to have potential mediation effects in the pathway from gut microbiota to obesity. If the identified metabolites, peripheral cells, or cytokines did not only exist in a triangular relationship but also had mediation effects significantly different from 0, they were considered as mediators with strong evidence.

## 3 Results

### 3.1 Causal effects of gut microbiota on obesity

**Figure 2:**
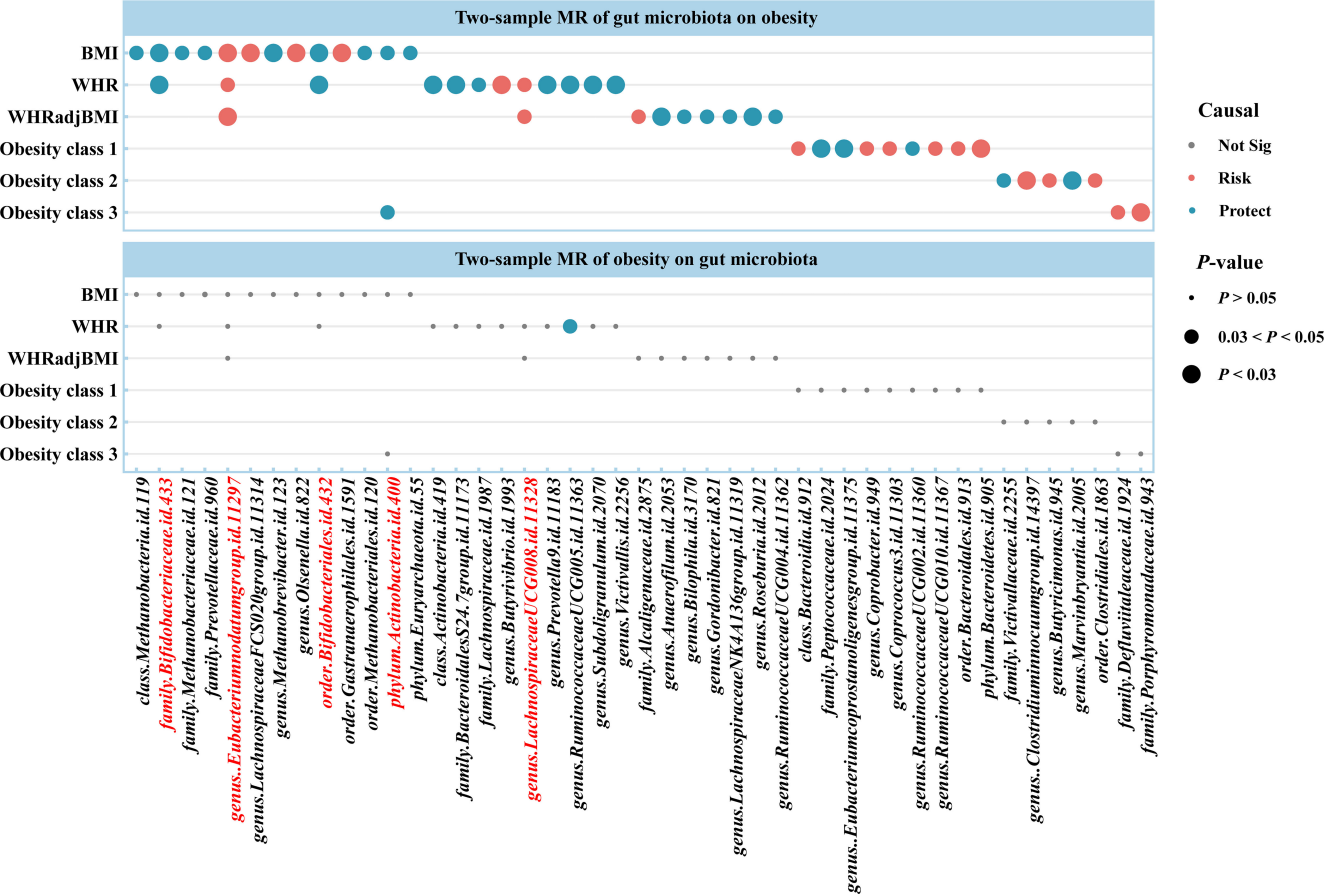
Causal estimates of bidirectional MR between gut microbiota and obesity. *Top*: Estimates from the IVW analysis of gut microbiota on obesity. *Bottom*: Estimates from the IVW analysis of obesity on gut microbiota. The bacterial features underlined in red were related to more than one obesity traits. BMI, body mass index; WHR, waist-to-hip ratio; WHRadjBMI, WHR adjusted for BMI; MR, Mendelian randomization; Not Sig, not significant.

Using two-sample MR, we identified 50 suggestive associations between gut microbiota and obesity (*P*_IVW_ < 0.05, FDR > 0.1; corresponding to 44 unique gut microbiota taxa). The causal microbial counts of the obesity traits BMI, WHR, WHRadjBMI, Obesity classes 1, 2, and 3 were 13, 12, 9, 9, 4, and 3, respectively (**Figure 2**; **Supplementary Table S3**). Five bacterial features were associated with more than one obesity trait, which may be a common molecular mechanism in the GWAS datasets of different obesity phenotypes. The phylum *Actinobacteria* (BMI; Obesity class 3), order *Bifidobacteriales* (BMI; WHR), and family *Bifidobacteriaceae* (BMI; WHR) had a negative causal relationship with obesity. In contrast the genus *Lachnospiraceae* UCG008 (WHR; WHRadjBMI) and species *Eubacterium nodatum* (BMI; WHR; WHRadjBMI) had a positive causal relationship with obesity (**Supplementary Figure S1**). *Lachnospiraceae* is closely related to obesity, and we found that the genus *Lachnospiraceae* FCS020 may increase the risk of obesity (BMI), whereas the family *Lachnospiraceae* and genus *Lachnospiraceae* NK4A136 may reduce the risk (WHRadjBMI). Moreover, the family belonged to the order subcategory; therefore, SNP sets included in families and their relevant orders might heavily overlap. These include SNPs of the family *Bifidobacteriales* and order *Bifidobacteriaceae* when applying MR analysis between the gut microbiota and obesity. Sensitivity analysis further verified the robustness of the MR results (**Supplementary Table S4**). The Q statistics showed no evidence of heterogeneity. Furthermore, the results of MR-Egger regression and MR-PRESSO analyses suggested no evidence of horizontal pleiotropy. Based on the MR-Steiger test, we did not find any reverse causality.

For causal associations between gut microbiota and obesity identified above, we conducted reverse MR and found a negative causal relationship between WHR and the genus *Ruminococcaceae* UCG005 (odds ratio [OR] = 0.877, 95% CI [0.776–0.992], *P* = 0.037) (**Figure 2**; **Supplementary Tables S5**, **S6**).

### 3.2 Causal effects of plasma metabolites on obesity

**Figure 3:**
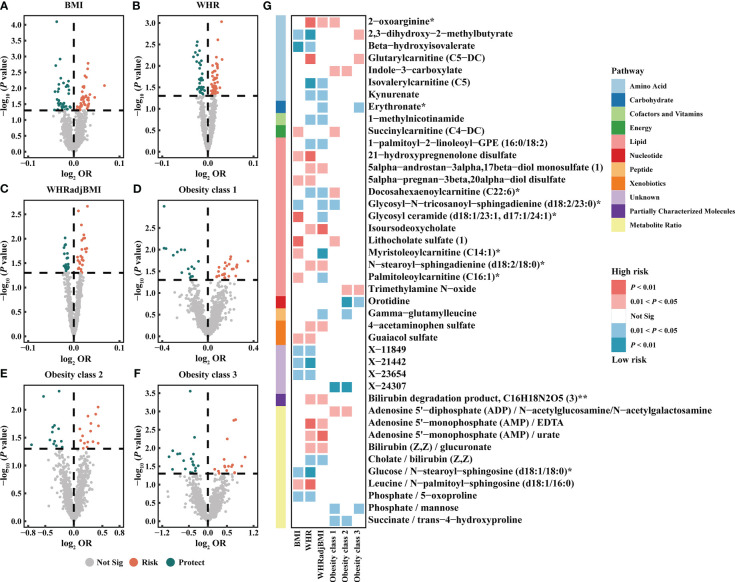
Causal estimates of plasma metabolites on obesity. **(A–F)** Volcano plots of the IVW MR for the associations between plasma metabolites and each obesity trait. **(G)** Heatmap of the 42 shared plasma metabolites that showed a causal association with more than one obesity trait at nominal significance (*P*_IVW_ < 0.05). BMI, body mass index; WHR, waist-to-hip ratio; WHRadjBMI, WHR adjusted for BMI; OR, odds ratio; Not Sig, not significant.

Based on the IVW method, the results suggested 327 causal relationships between plasma metabolomics and obesity (*P*_IVW_ < 0.05, corresponding to 281 unique plasma metabolites, 229 unique plasma metabolite levels, and 52 unique metabolic ratios). Among BMI, WHR, WHRadjBMI, Obesity classes 1, 2, and 3, there were 84 (73 metabolites and 11 ratios), 82 (67 metabolites and 15 ratios), 54 (44 metabolites and 10 ratios), 41 (34 metabolites and 7 ratios), 29 (20 metabolites and 9 ratios), and 37 (27 metabolites and 10 ratios) associations detected, respectively (**Figure 3**; **Supplementary Table S7**). Additionally, we observed 32 shared causal metabolites and 10 shared causal ratios for different obesity traits. Among them, plasma metabolites included lipids (13), amino acids (7), xenobiotics (2), nucleotide (1), cofactor and vitamins (1), carbohydrates (1), peptide (21), energy (1), partially characterized molecules (1), and unknown (4) (**Figure 3**). For example, 2-oxoarginine* had a positive causal relationship with WHR (OR = 1.0145, 95% CI [1.0035–1.0256], *P* = 0.0093), WHRadjBMI (OR = 1.0113, 95% CI [1.0001–1.0.225], *P* = 0.0471), and Obesity class 1 (OR = 1.1374, 95% CI [1.0190–1.2696], *P* = 0.0217). However, following FDR correction, only 1-(1-enyl-palmitoyl)-2-oleoyl-GPC (P-16:0/18:1)* maintained a significant negative causal relationship with BMI (OR = 0.9744, 95% CI [0.9619–0.9870], *P* = 0.0001, FDR = 0.0874) (**Supplementary Table S7**). These results were validated no heterogeneity and horizontal pleiotropy using sensitivity analyses (**Supplementary Table S8**).

The metabolic pathway analysis identified 17 significant metabolic pathways (12 unique pathways) (**Supplementary Figure S2**). We discovered metabolic pathways shared between different obesity phenotypes: “D-Arginine and D-ornithine metabolism” for WHR (*P* = 0.0332), WHRadjBMI (*P* = 0.0154), and Obesity class 1 (*P* = 0.0205); “Linoleic acid metabolism” for BMI (*P* = 0.0382), Obesity classes 1 (*P* = 0.0256), and 3 (*P* = 0.0192); “Glycerophospholipid metabolism” for BMI (*P* = 0.0299) and WHR (*P* = 0.0348) (**Supplementary Table S9**).

### 3.3 Causal effects of blood cells, peripheral immune cells, and inflammatory cytokines on obesity

**Figure 4:**
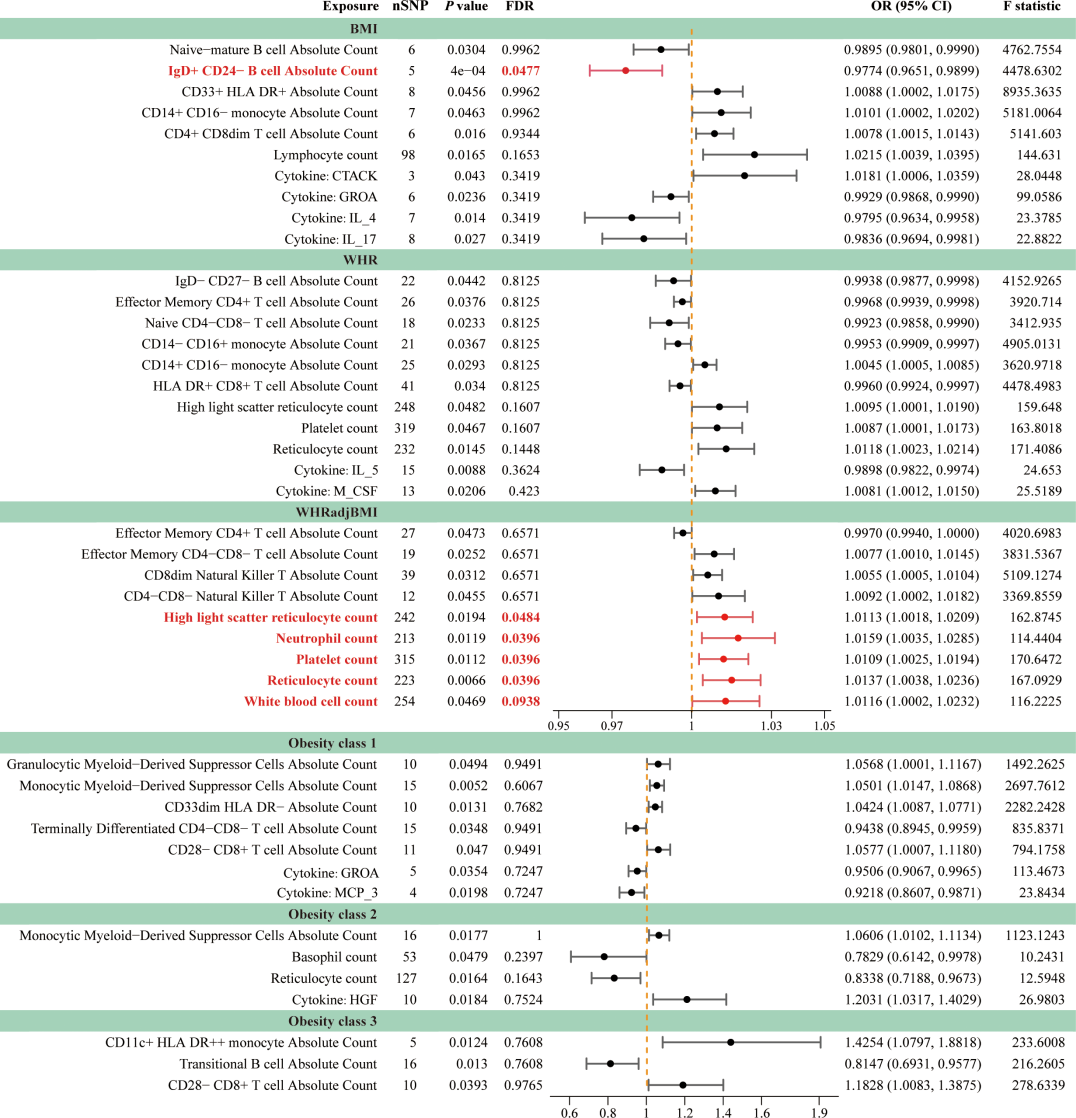
Forest plots for causal effects of peripheral cells and inflammatory cytokines on obesity. The horizontal bars correspond to the estimated OR with 95% CI using the IVW method for peripheral cells and inflammatory cytokines on obesity. Causal relationships that remain statistically significant after FDR correction were emphasized using red font and lines. FDR, false discovery rate; BMI, body mass index; WHR, waist-to-hip ratio; WHRadjBMI, WHR adjusted for BMI; OR, odds ratio; CI, confidence interval.

The IVW method revealed 35 associations between peripheral cells and obesity (*P*_IVW_ < 0.05, corresponding to 27 unique peripheral cells: 7 unique blood cells and 20 unique immune cells), including BMI, WHR, WHRadjBMI, Obesity classes 1, 2, and 3, with 6, 9, 9, 5, 3, and 3 associations, respectively (**Figure 4**; **Supplementary Table S10**). We identified seven shared causal cells, of which six cell traits had consistent causal effects among multiple obesity traits. The risk of obesity may be increased by two blood cell traits, high light scatter reticulocyte count (WHR, WHRadjBMI) and platelet count (WHR, WHRadjBMI), and three immune cell traits, *CD14^+^ CD16^–^* monocyte absolute count (BMI, WHR), *CD28^–^ CD8^+^* T cell absolute count (Obesity classes 1 and 3), and monocytic myeloid-derived suppressor cells absolute count (Obesity classes 1 and 2). One immune cell trait, effector memory *CD4^+^* T cell absolute count (WHR, WHRadjBMI), may reduce the risk of obesity. Next, we performed FDR correction on the results of the IVW method for blood cell and immune cell traits separately. The results indicate that after correction, five blood cell traits still exhibit a positive causal relationship with WHRadjBMI: high light scatter reticulocyte count (OR = 1.0113, 95% CI [1.0018–01.0209], *P* = 0.0194, FDR = 0.0484), neutrophil count (OR = 1.0159, 95% CI [1.0035–1.0285], *P* = 0.0119, FDR = 0.0396), platelet count (OR = 1.0109, 95% CI [1.0025–1.0194], *P* = 0.0112, FDR = 0.0396), reticulocyte count (OR = 1.0137, 95% CI [1.0038–1.0236], *P* = 0.0066, FDR = 0.0396), and white blood cell count (OR = 1.0116, 95% CI [1.0002–1.0232], *P* = 0.0469, FDR = 0.0938) (**Supplementary Table S10**). Additionally, the findings indicate that the immune cell trait *IgD^+^ CD24^–^* B cell absolute count continues to demonstrate a negative causal relationship with BMI (OR = 0.9774, 95% CI [0.9651–0.9899], *P* = 0.0004, FDR = 0.0477) (**Supplementary Table S10**). The MR results remained stable in the sensitivity analyses, suggesting the absence of significant heterogeneity and horizontal pleiotropy (**Supplementary Table S11**).

Moreover, the causal relationship between cytokines and obesity was evaluated using MR, and the results supported the existence of nine suggestive associations between cytokines and obesity (*P*_IVW_ < 0.05, FDR > 0.1; corresponding to eight unique cytokines), including four, four, two, and one association of BMI, WHR, Obesity classes 1, and 2, respectively (**Figure 4**; **Supplementary Table S12**). We found that growth-regulated alpha protein (GROA) (BMI: OR = 0.9929, 95% CI [0.9868–0.9990], *P* = 0.0236; Obesity class 1: OR = 0.9506, 95% CI [0.9067–0.9965], *P* = 0.0354) was the only shared causal cytokine that could reduce the risk of obesity. The sensitivity analyses further indicated the absence of heterogeneity and horizontal pleiotropy in these MR analyses (**Supplementary Table S13**).

### 3.4 Mediation analysis results

To explore the potential mechanisms of obesity occurrence and development, we conducted a mediation analysis to identify the causal pathway from gut microbiota to obesity mediated by plasma metabolites, peripheral cells, and inflammatory cytokines (please refer to the Mediation analysis section of Methods for details). This analysis focused on previously identified gut microbiota, metabolites, cells, and cytokines associated with obesity in the two-sample MR (**Supplementary Tables S3**, **S7**, **S10**, **S12**).

Firstly, the causal relationship among causal gut microbiota, metabolites, cells, and cytokines was evaluated via two-sample MR. We identified 95 associations of gut microbiota to metabolites (BMI, 41; WHR, 31; WHRadjBMI, 9; Obesity class 1, 8; Obesity class 2, 5; Obesity class 3, 1), 10 associations to cells (WHR, 2; WHRadjBMI, 4; Obesity class 1, 1; Obesity class 2, 1; Obesity class 3, 2), and 2 associations to cytokines (WHR, 1; Obesity class 1, 1) (**Supplementary Table S14**). Furthermore, MVMR analysis was used to screen for metabolites, cells, and cytokines that exhibit a causal relationship with obesity after correcting for the gut microbiota. The results showed that after microbial adjustment, there were 34 metabolite–obesity associations (BMI, 14; WHR, 14; WHRadjBMI, 5; Obesity class 2, 1), 7 cell–obesity associations (WHR, 2; WHRadjBMI, 4; Obesity class 2, 1), and 2 cytokine–obesity associations (WHR, 1; Obesity class 1, 1) (**Supplementary Figure S3**; **Supplementary Table S15**). These MR results were validated through the sensitivity analysis, further suggesting the absence of heterogeneity and horizontal pleiotropy (**Supplementary Tables S16**, **S17**).

In summary, we identified 20 mediating relationships (1 with strong evidence, 19 with potential evidence), including 17, 2, and 1 gut microbiota–obesity causal pathways mediated by metabolites, cells, and cytokines, respectively (**Table 1**). The mediation analysis reveals that only 1-(1-enyl-palmitoyl)-2-oleoyl-GPC (p-16:0/18:1) levels exhibit significant negative mediation effects (beta = −0.0043, 95% CI [−0.0085, −0.0001], *P* = 0.0462) on phylum *Actinobacteria* and BMI with 13.55% (95% CI: 0.23%, 26.87%) proportion. The pathway from phylum *Actinobacteria* to BMI was also potentially mediated by 1-(1-enyl palmitoyl)-2-palmitoyl-GPC (P-16:0/16:1) levels with 4.97% proportion. Additionally, three additional microbial features exhibited more than one mediator. The mediation ratios from class *Actinobacteria* to WHR through methionine sulfone and X-16935 (an unknown metabolite) levels were 12.29% and 15.06%, respectively. The mediating ratios of genus *Lachnospiraceae* UCG008 to WHR through 1-palmitoyl-2-oleoyl-GPE (16:0/18:1) and 1,2-dilinoleoyl-GPE (18:2/18:2) levels were 7.21% and 8.24%, respectively. The mediating ratios of family *Alcaliginaceae* to WHRadjBMI through gamma-glutamylvaline levels and high light scatter reticulocyte count were 12.89% and 2.15%, respectively. Two mediators mediated more than one relationship: metabolite 3-hydroxybutyrate levels mediated class *Metanobacteria*, order *Metanobacteriales*, and family *Metanobacteriaceae* to BMI, all with a mediation ratio of 10.74%. The ratios of myo-inositol levels mediated order *Bifidobacteriaceae* and family *Bifidobacteriaceae* to WHR (both 8.14%). White cells are a very important type of immune cell in human blood. The white blood cell counts mediated genus *Anaerofilum* to WHRadjBMI, with 1.87% proportion. GROA is a typical inflammatory chemokine with a mediating ratio of 8.64% between the genus *Ruminococcaceae* UCG010 and Obesity class 1. These results showed the consistent direction of the total, indirect, and direct effects, and that the leave-one-out analysis supported the reliable causal relationship in the two-sample MR study of exposure to outcome, exposure to mediator, and mediator to outcome (**Table 1**; **Supplementary Figure S4**; **Supplementary Table S18**).

**Table 1:** Mediation effect of gut microbiota on obesity via plasma metabolites, peripheral cells and inflammatory cytokines.

Exposure	Mediator	Outcome	Total effect	Direct effect	Mediation effect (95% CI)	*P*-value	Mediation Proportion (95% CI) #
*p_Actinobacteria*	1-(1-enyl-palmitoyl)-2-oleoyl-GPC (P-16:0/18:1)*	BMI	-0.0318	-0.0275	**-0.0043 (-0.0085, -0.0001)**	**0.0462**	**13.55%** **(0.23%, 26.87%)**
*p_Actinobacteria*	1-(1-enyl-palmitoyl)-2-palmitoleoyl-GPC (P-16:0/16:1)*	BMI	-0.0318	-0.0302	-0.0016 (-0.0036, 0.0004)	0.1171	4.97%
*c_Methanobacteria*	3-hydroxybutyrate	BMI	-0.0149	-0.0133	-0.0016 (-0.0039, 0.0007)	0.1696	10.74%
*o_Methanobacteriales*	3-hydroxybutyrate	BMI	-0.0149	-0.0133	-0.0016 (-0.0039, 0.0007)	0.1696	10.74%
*f_Methanobacteriaceae*	3-hydroxybutyrate	BMI	-0.0149	-0.0133	-0.0016 (-0.0039, 0.0007)	0.1696	10.74%
*c_Actinobacteria*	methionine sulfone	WHR	-0.0189	-0.0166	-0.0023 (-0.005, 0.0004)	0.0896	12.29%
*c_Actinobacteria*	X-16935	WHR	-0.0189	-0.0161	-0.0028 (-0.006, 0.0003)	0.0784	15.06%
*o_Bifidobacteriales*	myo-inositol	WHR	-0.0196	-0.018	-0.0016 (-0.0039, 0.0007)	0.1783	8.14%
*f_Bifidobacteriaceae*	myo-inositol	WHR	-0.0196	-0.018	-0.0016 (-0.0039, 0.0007)	0.1783	8.14%
*f_Lachnospiraceae*	deoxycarnitine	WHR	-0.0215	-0.0198	-0.0017 (-0.0038, 0.0004)	0.1175	7.85%
*g_Lachnospiraceae* UCG008	1-palmitoyl-2-oleoyl-GPE (16:0/18:1)	WHR	0.016	0.0148	0.0012 (-0.0005, 0.0028)	0.1592	7.21%
*g_Lachnospiraceae* UCG008	1,2-dilinoleoyl-GPE (18:2/18:2)*	WHR	0.016	0.0147	0.0013 (-0.0006, 0.0032)	0.1765	8.24%
*g_Subdoligranulum*	docosapentaenoate (n3 DPA; 22:5n3)	WHR	-0.0292	-0.0259	-0.0032 (-0.0072, 0.0008)	0.1121	11.11%
*g_Victivallis*	X-13431	WHR	-0.0169	-0.0162	-0.0008 (-0.0016, 0.0001)	0.0761	4.51%
*f_Alcaligenaceae*	gamma-glutamylvaline	WHRadjBMI	0.0192	0.0168	0.0025 (-0.0008, 0.0058)	0.1443	12.89%
*f_Alcaligenaceae*	high light scatter reticulocyte count	WHRadjBMI	0.0192	0.0188	0.0004 (-4.52E-05, 0.0009)	0.0773	2.15%
*g_Lachnospiraceae* NK4A136	pimeloylcarnitine/3-methyladipoylcarnitine (C7-DC)	WHRadjBMI	-0.0165	-0.0147	-0.0018 (-0.0041, 0.0005)	0.1289	10.88%
*s_Eubacterium nodatum*	glycosyl-N-palmitoyl-sphingosine (d18:1/16:0)	WHRadjBMI	0.011	0.0096	0.0013 (-0.0004, 0.0030)	0.1261	12.14%
*g_Anaerofilum*	white blood cell count	WHRadjBMI	-0.0147	-0.0144	-0.0003 (-0.0006, 1.30E-05)	0.0613	1.87%
*g_Ruminococcaceae* UCG010	GROA	Obesity class 1	0.2152	0.1966	0.0186 (-0.0054, 0.0425)	0.1280	8.64%

**#** When the 95% CI of the mediation effect spans 0, the 95% CI for mediation proportion is not calculated, as the direction of the upper or lower limit of the mediation effect is opposite to the total effect. Bold formatting indicates that the *P*-value is less than 0.05. BMI, body mass index; WHR, waist-to-hip ratio; WHRadjBMI, WHR adjusted for BMI; CI, confidence interval.

## 4 Discussion

This study comprehensively evaluated the causal relationship among gut microbiota, plasma metabolome, blood cells, peripheral immune cells, inflammatory cytokines, and obesity using MR analysis. We found potential causal associations between 44 bacterial features, 281 plasma metabolites (229 metabolites and 52 ratios), 27 peripheral cells (7 blood and 20 immune cells), and 8 inflammatory cytokines and obesity. Pathway analysis of known plasma metabolites indicated that D-arginine, D-ornithine, linoleic acid, and glycerophospholipid metabolism play important roles in the occurrence and development of obesity. In addition, the mediation analysis results supported the mediating effects of plasma metabolites, peripheral cells, and inflammatory cytokines on the gut microbiota in obesity pathogenesis.

Our findings suggest that higher bacterial abundance within phylum *Actinobacteria*, order *Bifidobacteriales* (the subcategory of phylum *Actinobacteria*), and family *Bifidobacteriaceae* (the subcategory of order *Bifidobacteriales*), may confer protection against obesity (48, 49). The abundance of *Bifidobacterium* (a subcategory of the family *Bifidobacteriaceae*) decreased significantly in individuals with increased visceral adipose tissue, BMI, blood triglycerides, and fatty liver (49). Obese patients can reduce their total blood sugar after short-term *Bifidobacterium*-based probiotic treatment and adjust the gut microbiota structure by increasing beneficial and decreasing pathogenic or opportunistic bacteria (50). In addition, many studies have shown that some strains of *Bifidobacterium* can function as probiotics to protect against obesity (51), such as *Bifidobacterium animalis subsp. Lactis* GCL2505, *Bifidobacterium breve* strain B-3, *Bifidobacterium breve* BR03, and *Bifidobacterium breve* B632 strains (52–54). *Bifidobacterium* can absorb sugars and produce short-chain fatty acids, especially acetate, which modulates host energy metabolism (e.g., inhibits fat accumulation in adipose tissue, increases insulin sensitivity, and enhances fatty acid / glucose metabolism) via the short-chain fatty acid receptor, G protein-coupled receptor 43, which is a common mechanism of probiotic activity (52).

The family *Lachnospiraceae* (phylum *Firmicutes*, class *Clostridia*) is one of the most important families of the intestinal microbiota in healthy adults, including 58 genera and several unclassified strains with complex functions and controversial roles in diseases (55, 56). Most human and mouse studies have revealed that an increased abundance of *Lachnospiraceae* is associated with metabolic diseases (57); however, certain controversies remain. For instance, some reports indicate positive and negative correlations between *Lachnospiraceae* ND3007, *Lachnospiraceae* NK4A136, and obesity, respectively (58). *Lachnospiraceae bacterium* 3_1_57FAA_cT1 is a potentially beneficial microorganism that is inversely proportional to homeostatic model assessment of insulin resistance (HOMA-IR) and fasting insulin levels and may mediate the impact of obesity on insulin resistance (59). The beneficial effects of *Lachnospiraceae* NK4A136 and *Lachnospiraceae bacterium* 3_1_57FAA_cT1 can be explained by the production of butyrate in the intestine (56, 58, 59). The genus *Lachnospiraceae* UCG008 emerged as a shared-risk bacterium across multiple obesity-related traits, consistent with its association with an elevated risk of various diseases, such as hemorrhagic stroke and periodontitis (60–62). Our findings suggest that genera *Lachnospirace* UG008 and *Lachnospirace* FCS020 may increase the risk of obesity, while the family *Lachnospiraceae* and genus *Lachnospiraceae* NK4A136 may reduce this risk.

Contrary to a previous study highlighting statistical associations between *Enterobacteriaceae*, *Desulfovibrio*, and *Akkermansia* with obesity (11), our MR results did not support these findings. This disparity in interpretation may be partly attributed to residual confounding and reverse causation observed in observational studies, rather than a validated causal correlation. It’s worth noting that the gut microbiota encompasses not only the bacteriome but also the mycobiome and virome, both of which contribute to obesity pathogenesis (63, 64). While our present study solely focuses on the gut bacteriome, future research will explore the relationship between fungi, viruses, and obesity. Nonetheless, controversies persist regarding the relationship between gut microbiota and obesity (65).

We found that D-arginine, D-ornithine, linoleic acid, and glycophospholipid metabolism were key pathways associated with obesity. Previous studies have shown that these metabolic pathways are mainly affected by gestational diabetes (66) and that D-arginine and D-ornithine metabolism are the main pathways associated with perinatal obesity (67). Linoleic acid is an omega-6 polyunsaturated fatty acid commonly found in the diet and is crucial for human health. Moderate intake of linoleic acid has a positive effect on maintaining cell membrane health and nervous system function. However, a high intake of linoleic acid may contribute to the obesity epidemic as the rich content of omega-6 fatty acids in modern diets is generally imbalanced by the intake of omega-3 fatty acids. Moreover, linoleic acid is converted into arachidonic acid in the body, which plays a role in inducing inflammation and fat synthesis (68, 69). Glycerophospholipid metabolism involves the synthesis, degradation, and remodeling of glycerophospholipids. Glycerophospholipids are the most abundant phospholipids in mammalian cell membranes and can be divided into subcategories, such as phosphatidylcholine (PC), phosphatidylethanolamine (PE), and phosphatidylserine. Animal studies have shown that abnormal levels and proportions of PC and PE can lead to dyslipidemia (70), obesity (71), and insulin resistance (72). Human studies have also shown that PC and PE are associated with T2D (73) and the risk of metabolic syndrome (74).

Myo-inositol is a sugar alcohol containing six carbon atoms that helps improve insulin sensitivity, and its deficiency may be related to the pathogenesis of metabolic diseases, such as metabolic syndrome, polycystic ovary syndrome, and diabetes (75). Myo-inositol has potential therapeutic effects on metabolic diseases (76). Our study supports a negative causal relationship between myo-inositol and obesity. Additionally, the mediation ratio of the myo-inositol-mediated order *Bifidobacteriales* and family *Bifidobacteriaceae* to the obesity trait WHR was 8.14%. 3-Hydroxybutyrate is a normal metabolic product of fatty acid oxidation and can be used as an energy source without sufficient blood sugar. It is also an important regulatory molecule that can affect gene expression, lipid metabolism, neuronal function, and the overall metabolic rate (77). The levels of 3-hydroxybutyrate are high in obese patients and decrease after weight loss surgery (78, 79). Our study supports a positive causal relationship between 3-hydroxybutyrate and obesity. Mediation analysis showed that the mediating proportion of the 3-hydroxybutyrate mediating class *Methanobacteria*, order *Methanobacteriales*, and family *Methanobacteriaceae* to the obesity trait BMI was 10.74%.

Obesity is a chronic inflammatory disease, and an increase in the white blood cell count has been widely associated with these diseases. Observational research has shown that the white blood cell count is positively correlated with the incidence of diabetes, hypertension, obesity, dyslipidemia, and metabolic syndrome (19). White blood cell count is a marker of inflammation and an indicator of whether obesity increases the risk of T2D. A high white blood cell count is associated with reduced insulin sensitivity (80, 81). This study indicated a positive causal relationship between white blood cell counts and obesity, and white blood cell counts mediated 1.87% of the effect of genus *Anaerofilum* on obesity trait WHRadjBMI. The pro-inflammatory cytokine GROA, also known as C-X-C motif chemokine ligand 1 (CXCL1), is a CXC chemotactic factor that helps in the recruitment and migration of various immune cells and plays an important role in regulating immune and inflammatory responses (82). Previous studies have shown that an increase in serum CXCL1 is associated with obesity, hyperglycemia, and pancreatic dysfunction (83). However, using MR, we found that CXCL1 may reduce the risk of obesity. In addition, a mediating ratio of 8.64% was observed for the CXCL1, which mediated genus *Ruminococcaceae* UCG010 to the obesity trait Obesity class 1.

This is the first time that a comprehensive MR framework has been used to analyze the causal relationship among gut microbiota, plasma metabolites, blood cells, peripheral immune cells, inflammatory cytokines, and obesity. Furthermore, a pathway from the gut microbiota to obesity was constructed through a two-step MR and mediation analysis via plasma metabolites, blood cells, peripheral immune cells, and inflammatory factors. This study used a series of sensitivity analyses to maximize the robustness of the MR results. However, this study has certain limitations. First, the lack of demographic information, such as age and sex, in the initial study hindered further subgroup analyses. Second, the majority of people studied by the GWAS were of European ancestry; therefore, the generalizability of the research results to other populations is limited. In addition, although the MR method is effective in evaluating the causal relationship between exposure factors and outcomes, this result needs to be further validated based on more experimental and clinical studies.

## 5 Conclusion

In summary, our MR study identified 44 gut microbiota taxa, 281 plasma metabolites, 27 peripheral cells, and 8 inflammatory cytokines that were causally linked to obesity; among them, 5 shared bacterial features, 42 shared metabolites, 7 shared cells, and 1 shared cytokine. Pathway analysis revealed 12 obesity-related metabolic pathways, with particular emphasis on D-arginine, D-ornithine, linoleic acid, and glycerophospholipid metabolism which were closely related to obesity. Moreover, we found 20 mediating relationships, including the causal pathways mediated by 17 metabolites, 2 peripheral cells, and 1 inflammatory cytokine from gut microbiota to obesity.

This MR analysis supports the causal effects of the gut microbiota, plasma metabolites, peripheral cells, and inflammatory cytokines on obesity. In addition, mediation analysis revealed that plasma metabolites, peripheral cells, and inflammatory cytokines mediate the pathway from the gut microbiota to obesity. The identified gut microbiota, plasma metabolites, and cellular and inflammatory factors may serve as biomarkers for the diagnosis and treatment of obesity and contribute to the study of obesity mechanisms.

## Data availability statement

The original contributions presented in the study are included in the article/**Supplementary Material**. Further inquiries can be directed to the corresponding authors.

## Ethics statement

All analyses were based on publicly available summary statistics, which do not require ethical approval and consent.

## Author contributions

YL: Investigation, Methodology, Software, Writing – original draft, Formal analysis. XW: Methodology, Software, Validation, Visualization, Writing – original draft, Formal analysis. ZZ: Data curation, Resources, Writing – original draft. LS: Conceptualization, Funding acquisition, Methodology, Supervision, Writing – original draft, Project administration. LC: Conceptualization, Methodology, Writing – review and editing. XZ: Conceptualization, Funding acquisition, Writing – review and editing.
