# Supplementary material for: Effect of the gut microbiome, plasma metabolome, peripheral cells, and inflammatory cytokines on obesity: a bidirectional two-sample Mendelian randomization study and mediation analysis
Source: Front Immunol. 2024 Mar 15;15:1348347. doi: 10.3389/fimmu.2024.1348347 (PMC10981273; doi:10.3389/fimmu.2024.1348347)
Supplement: Supplementary file 1 [file DataSheet_1.docx]

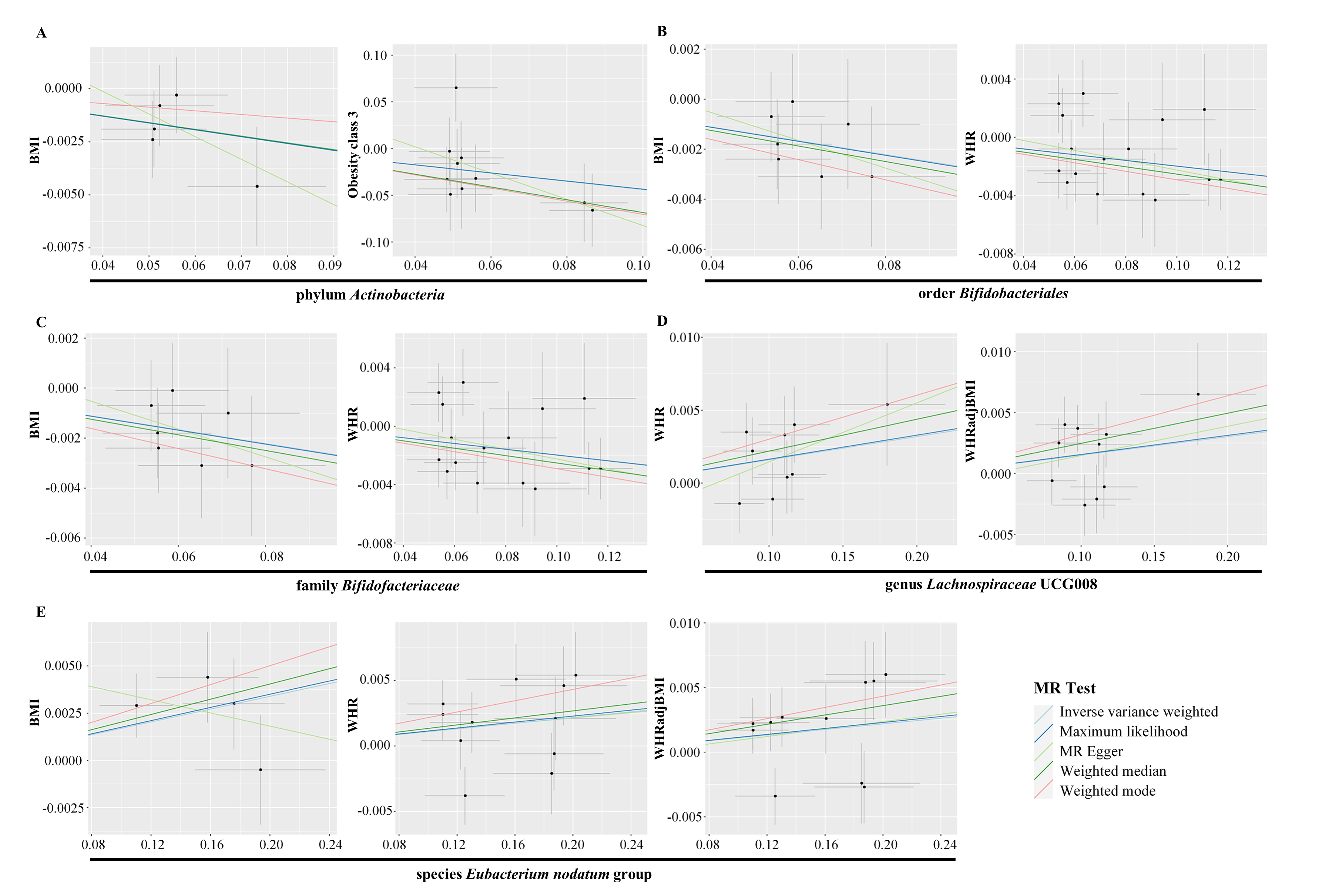


**Figure S1 Scatter plots for the causal associations between five shared bacterial features on obesity.** Each black point represents the SNP effect sizes on the exposure (horizontal axis) and outcome (vertical axis) and is plotted with error bars of standard error (SE). The line slopes represent the causal association for each method: the inverse variance weighted (light blue line), maximum likelihood (blue line), MR-Egger regression (light green line), weighted median (green line), and weighted mode (red line).


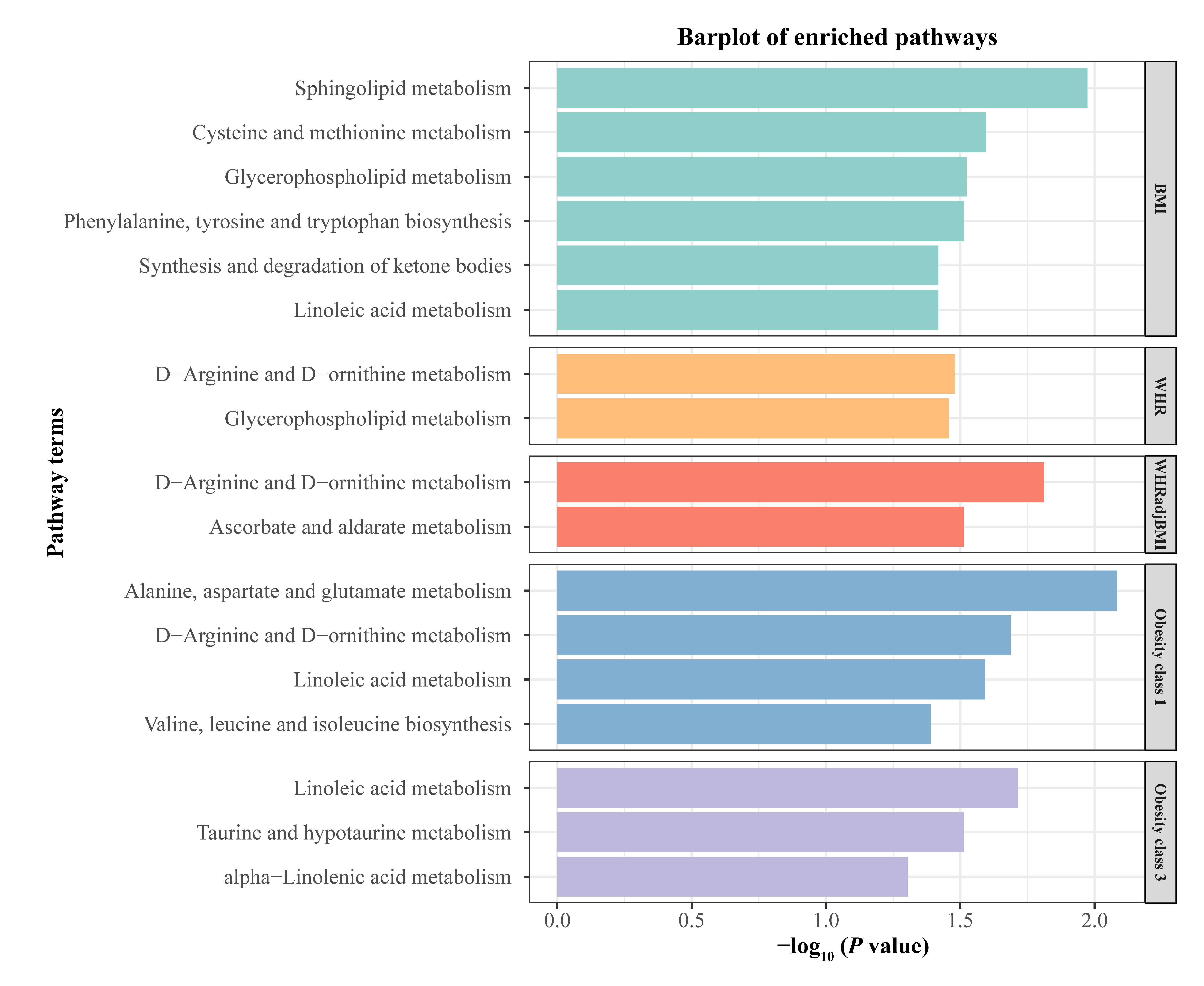


**Figure S2 Enriched significant metabolic pathways of obesity-associated metabolites.**





**Figure S3 Forest plots for MVMR analyses of plasma metabolites, peripheral cells, and inflammatory cytokines on obesity after adjusting for gut microbiota.**
