# Supplementary material for: Effect of the gut microbiome, plasma metabolome, peripheral cells, and inflammatory cytokines on obesity: a bidirectional two-sample Mendelian randomization study and mediation analysis
Source: Front Immunol. 2024 Mar 15;15:1348347. doi: 10.3389/fimmu.2024.1348347 (PMC10981273; doi:10.3389/fimmu.2024.1348347)
Supplement: Supplementary file 2 [file DataSheet_2.pdf]

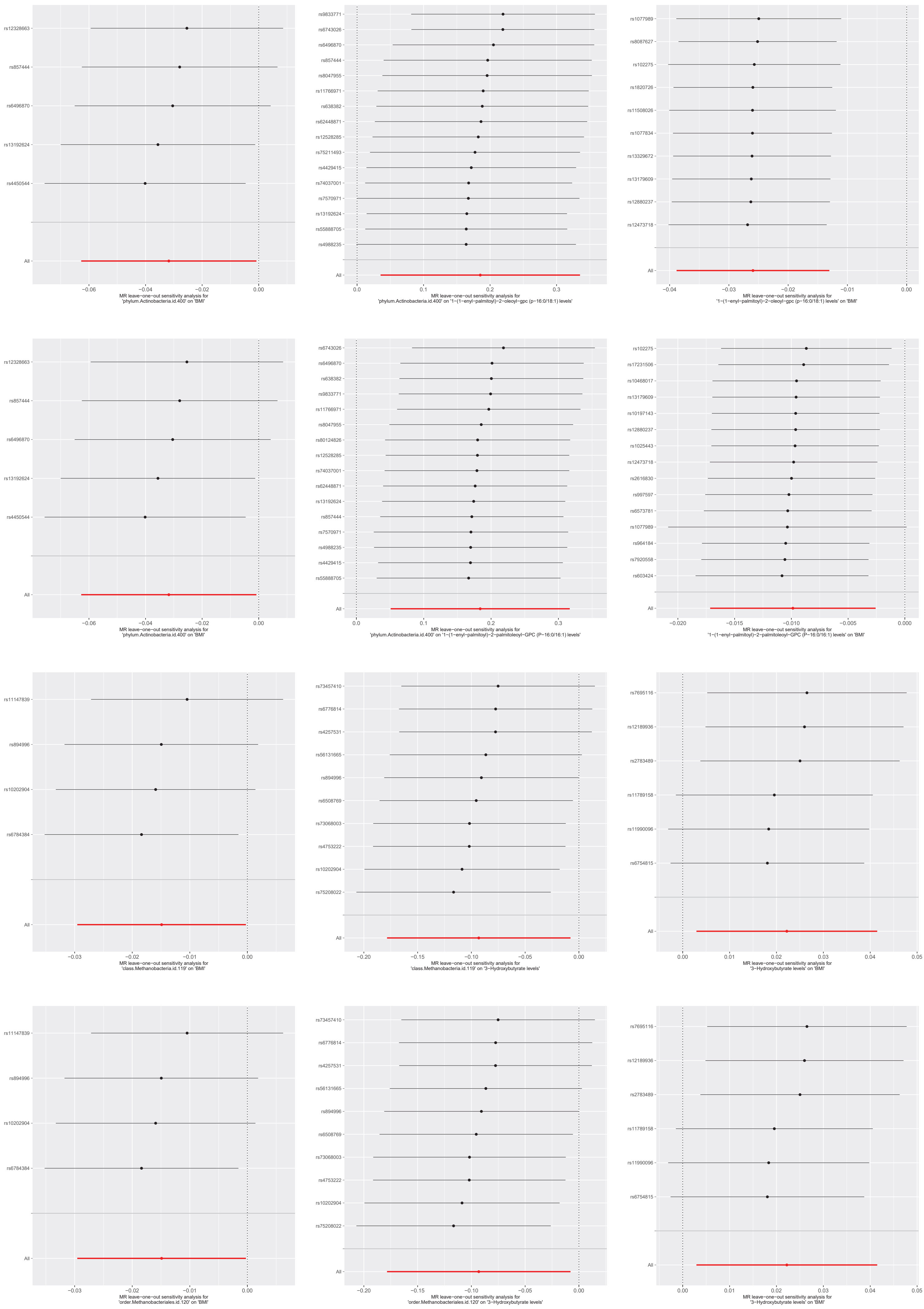
rs12328663
rs857444
rs6496870
rs13192624
rs4450544
All
−0.06 −0.04 −0.02 0.00
MR leave−one−out sensitivity analysis for
'phylum.Actinobacteria.id.400' on 'BMI'
rs9833771
rs6743026
rs6496870
rs857444
rs8047955
rs11766971
rs638382
rs62448871
rs12528285
rs75211493
rs4429415
rs74037001
rs7570971
rs13192624
rs55888705
rs4988235
All
0.0 0.1 0.2 0.3
MR leave−one−out sensitivity analysis for
'phylum.Actinobacteria.id.400' on '1−(1−enyl−palmitoyl)−2−oleoyl−gpc (p−16:0/18:1) levels'
rs1077989
rs8087627
rs102275
rs1820726
rs11508026
rs1077834
rs13329672
rs13179609
rs12880237
rs12473718
All
−0.04 −0.03 −0.02 −0.01 0.00
MR leave−one−out sensitivity analysis for
'1−(1−enyl−palmitoyl)−2−oleoyl−gpc (p−16:0/18:1) levels' on 'BMI'
rs12328663
rs857444
rs6496870
rs13192624
rs4450544
All
−0.06 −0.04 −0.02 0.00
MR leave−one−out sensitivity analysis for
'phylum.Actinobacteria.id.400' on 'BMI'
rs6743026
rs6496870
rs638382
rs9833771
rs11766971
rs8047955
rs80124826
rs12528285
rs74037001
rs62448871
rs13192624
rs857444
rs7570971
rs4988235
rs4429415
rs55888705
All
0.0 0.1 0.2 0.3
MR leave−one−out sensitivity analysis for
'phylum.Actinobacteria.id.400' on '1−(1−enyl−palmitoyl)−2−palmitoleoyl−GPC (P−16:0/16:1) levels'
rs102275
rs17231506
rs10468017
rs13179609
rs10197143
rs12880237
rs1025443
rs12473718
rs2616830
rs997597
rs6573781
rs1077989
rs964184
rs7920558
rs603424
All
−0.020 −0.015 −0.010 −0.005 0.000
MR leave−one−out sensitivity analysis for
'1−(1−enyl−palmitoyl)−2−palmitoleoyl−GPC (P−16:0/16:1) levels' on 'BMI'
rs11147839
rs894996
rs10202904
rs6784384
All
−0.03 −0.02 −0.01 0.00
MR leave−one−out sensitivity analysis for
'class.Methanobacteria.id.119' on 'BMI'
rs73457410
rs6776814
rs4257531
rs56131665
rs894996
rs6508769
rs73068003
rs4753222
rs10202904
rs75208022
All
−0.20 −0.15 −0.10 −0.05 0.00
MR leave−one−out sensitivity analysis for
'class.Methanobacteria.id.119' on '3−Hydroxybutyrate levels'
rs7695116
rs12189936
rs2783489
rs11789158
rs11990096
rs6754815
All
0.00 0.01 0.02 0.03 0.04 0.05
MR leave−one−out sensitivity analysis for
'3−Hydroxybutyrate levels' on 'BMI'
rs11147839
rs894996
rs10202904
rs6784384
All
−0.03 −0.02 −0.01 0.00
MR leave−one−out sensitivity analysis for
'order.Methanobacteriales.id.120' on 'BMI'
rs73457410
rs6776814
rs4257531
rs56131665
rs894996
rs6508769
rs73068003
rs4753222
rs10202904
rs75208022
All
−0.20 −0.15 −0.10 −0.05 0.00
MR leave−one−out sensitivity analysis for
'order.Methanobacteriales.id.120' on '3−Hydroxybutyrate levels'
rs7695116
rs12189936
rs2783489
rs11789158
rs11990096
rs6754815
All
0.00 0.01 0.02 0.03 0.04 0.05
MR leave−one−out sensitivity analysis for
'3−Hydroxybutyrate levels' on 'BMI'

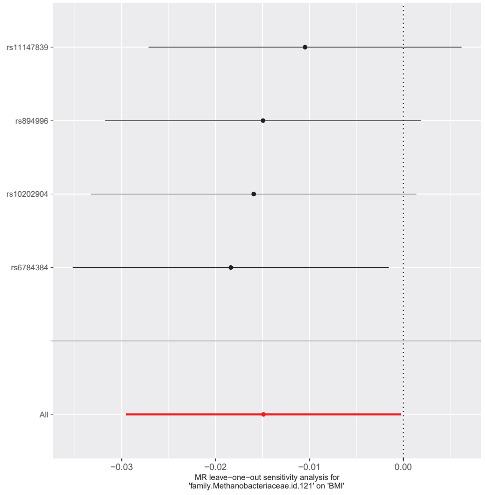
rs11147839
rs894996
rs10202904
rs6784384
All
−0.03 −0.02 −0.01 0.00
MR leave−one−out sensitivity analysis for
'family.Methanobacteriaceae.id.121' on 'BMI'

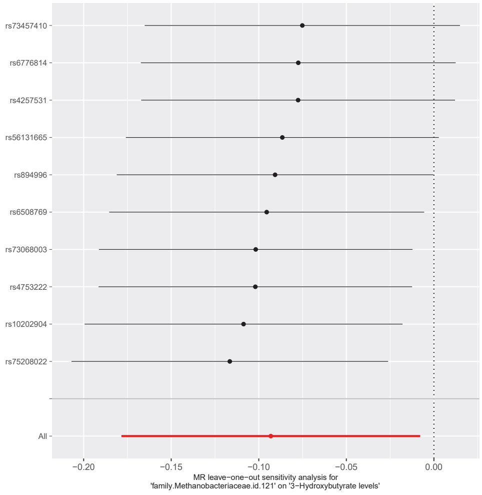
rs73457410
rs6776814
rs4257531
rs56131665
rs894996
rs6508769
rs73068003
rs4753222
rs10202904
rs75208022
All
−0.20 −0.15 −0.10 −0.05 0.00
MR leave−one−out sensitivity analysis for
'family.Methanobacteriaceae.id.121' on '3−Hydroxybutyrate levels'

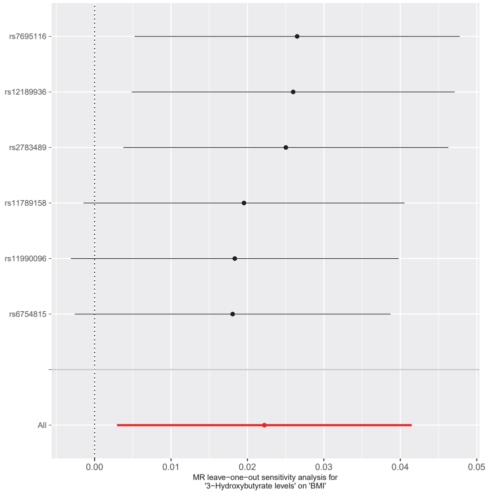
rs7695116
rs12189936
rs2783489
rs11789158
rs11990096
rs6754815
All
0.00 0.01 0.02 0.03 0.04 0.05
MR leave−one−out sensitivity analysis for
'3−Hydroxybutyrate levels' on 'BMI'

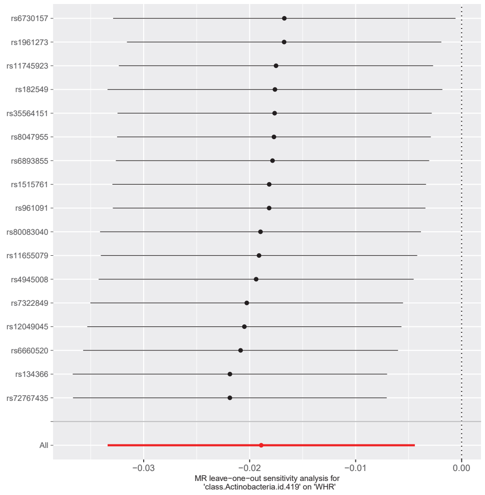
rs6730157
rs1961273
rs11745923
rs182549
rs35564151
rs8047955
rs6893855
rs1515761
rs961091
rs80083040
rs11655079
rs4945008
rs7322849
rs12049045
rs6660520
rs134366
rs72767435
All
−0.03 −0.02 −0.01 0.00
MR leave−one−out sensitivity analysis for
'class.Actinobacteria.id.419' on 'WHR'

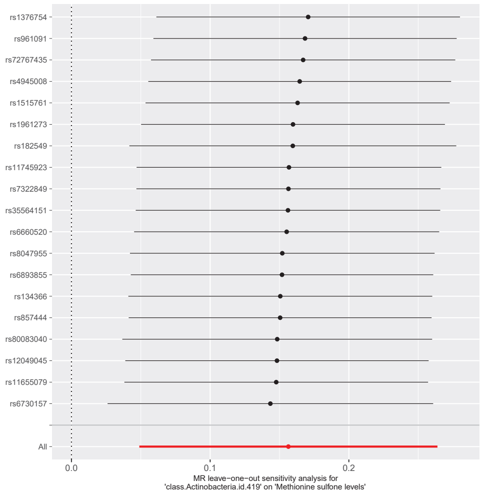
rs1376754
rs961091
rs72767435
rs4945008
rs1515761
rs1961273
rs182549
rs11745923
rs7322849
rs35564151
rs6660520
rs8047955
rs6893855
rs134366
rs857444
rs80083040
rs12049045
rs11655079
rs6730157
All
0.0 0.1 0.2
MR leave−one−out sensitivity analysis for
'class.Actinobacteria.id.419' on 'Methionine sulfone levels'

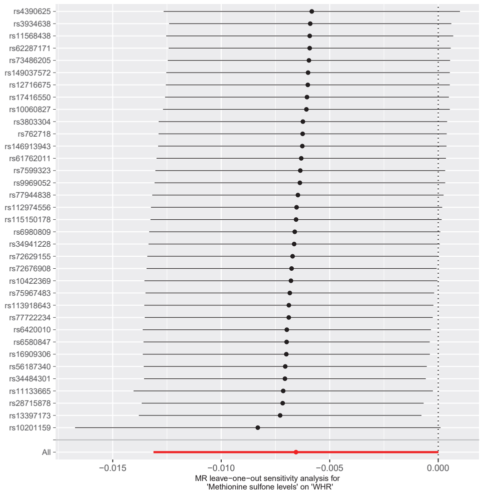
rs4390625
rs3934638
rs11568438
rs62287171
rs73486205
rs149037572
rs12716675
rs17416550
rs10060827
rs3803304
rs762718
rs146913943
rs61762011
rs7599323
rs9969052
rs77944838
rs112974556
rs115150178
rs6980809
rs34941228
rs72629155
rs72676908
rs10422369
rs75967483
rs113918643
rs77722234
rs6420010
rs6580847
rs16909306
rs56187340
rs34484301
rs11133665
rs28715878
rs13397173
rs10201159
All
−0.015 −0.010 −0.005 0.000
MR leave−one−out sensitivity analysis for
'Methionine sulfone levels' on 'WHR'

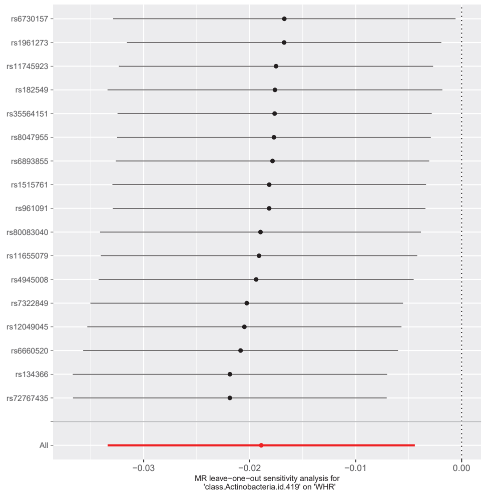
rs6730157
rs1961273
rs11745923
rs182549
rs35564151
rs8047955
rs6893855
rs1515761
rs961091
rs80083040
rs11655079
rs4945008
rs7322849
rs12049045
rs6660520
rs134366
rs72767435
All
−0.03 −0.02 −0.01 0.00
MR leave−one−out sensitivity analysis for
'class.Actinobacteria.id.419' on 'WHR'

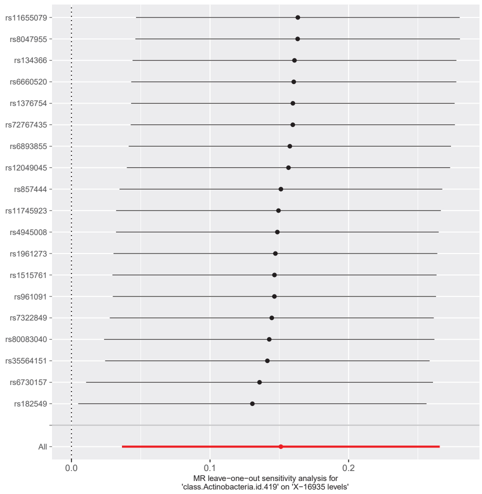
rs11655079
rs8047955
rs134366
rs6660520
rs1376754
rs72767435
rs6893855
rs12049045
rs857444
rs11745923
rs4945008
rs1961273
rs1515761
rs961091
rs7322849
rs80083040
rs35564151
rs6730157
rs182549
All
0.0 0.1 0.2
MR leave−one−out sensitivity analysis for
'class.Actinobacteria.id.419' on 'X−16935 levels'

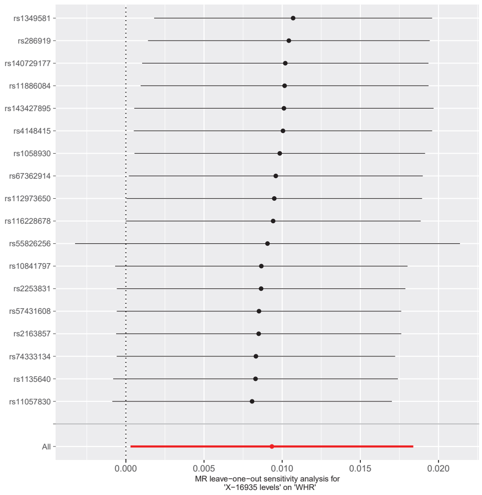
rs1349581
rs286919
rs140729177
rs11886084
rs143427895
rs4148415
rs1058930
rs67362914
rs112973650
rs116228678
rs55826256
rs10841797
rs2253831
rs57431608
rs2163857
rs74333134
rs1135640
rs11057830
All
0.000 0.005 0.010 0.015 0.020
MR leave−one−out sensitivity analysis for
'X−16935 levels' on 'WHR'

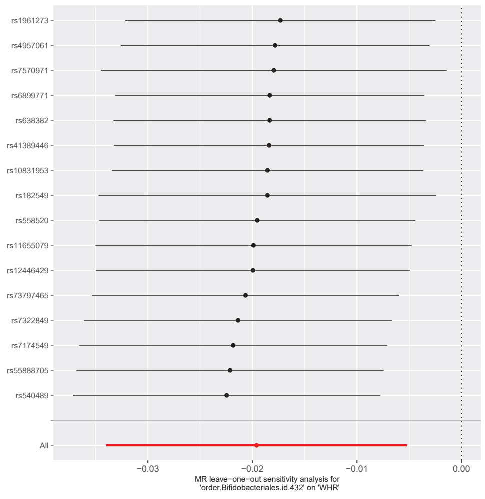
rs1961273
rs4957061
rs7570971
rs6899771
rs638382
rs41389446
rs10831953
rs182549
rs558520
rs11655079
rs12446429
rs73797465
rs7322849
rs7174549
rs55888705
rs540489
All
−0.03 −0.02 −0.01 0.00
MR leave−one−out sensitivity analysis for
'order.Bifidobacteriales.id.432' on 'WHR'

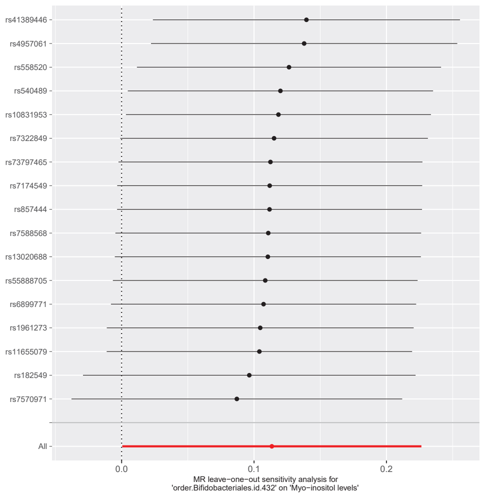
rs41389446
rs4957061
rs558520
rs540489
rs10831953
rs7322849
rs73797465
rs7174549
rs857444
rs7588568
rs13020688
rs55888705
rs6899771
rs1961273
rs11655079
rs182549
rs7570971
All
0.0 0.1 0.2
MR leave−one−out sensitivity analysis for
'order.Bifidobacteriales.id.432' on 'Myo−inositol levels'

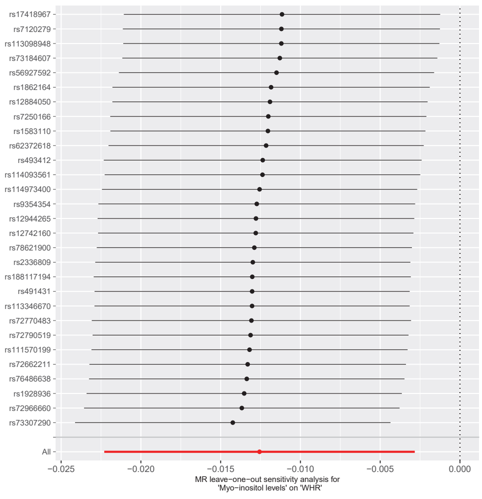
rs17418967
rs7120279
rs113098948
rs73184607
rs56927592
rs1862164
rs12884050
rs7250166
rs1583110
rs62372618
rs493412
rs114093561
rs114973400
rs9354354
rs12944265
rs12742160
rs78621900
rs2336809
rs188117194
rs491431
rs113346670
rs72770483
rs72790519
rs111570199
rs72662211
rs76486638
rs1928936
rs72966660
rs73307290
All
−0.025 −0.020 −0.015 −0.010 −0.005 0.000
MR leave−one−out sensitivity analysis for
'Myo−inositol levels' on 'WHR'

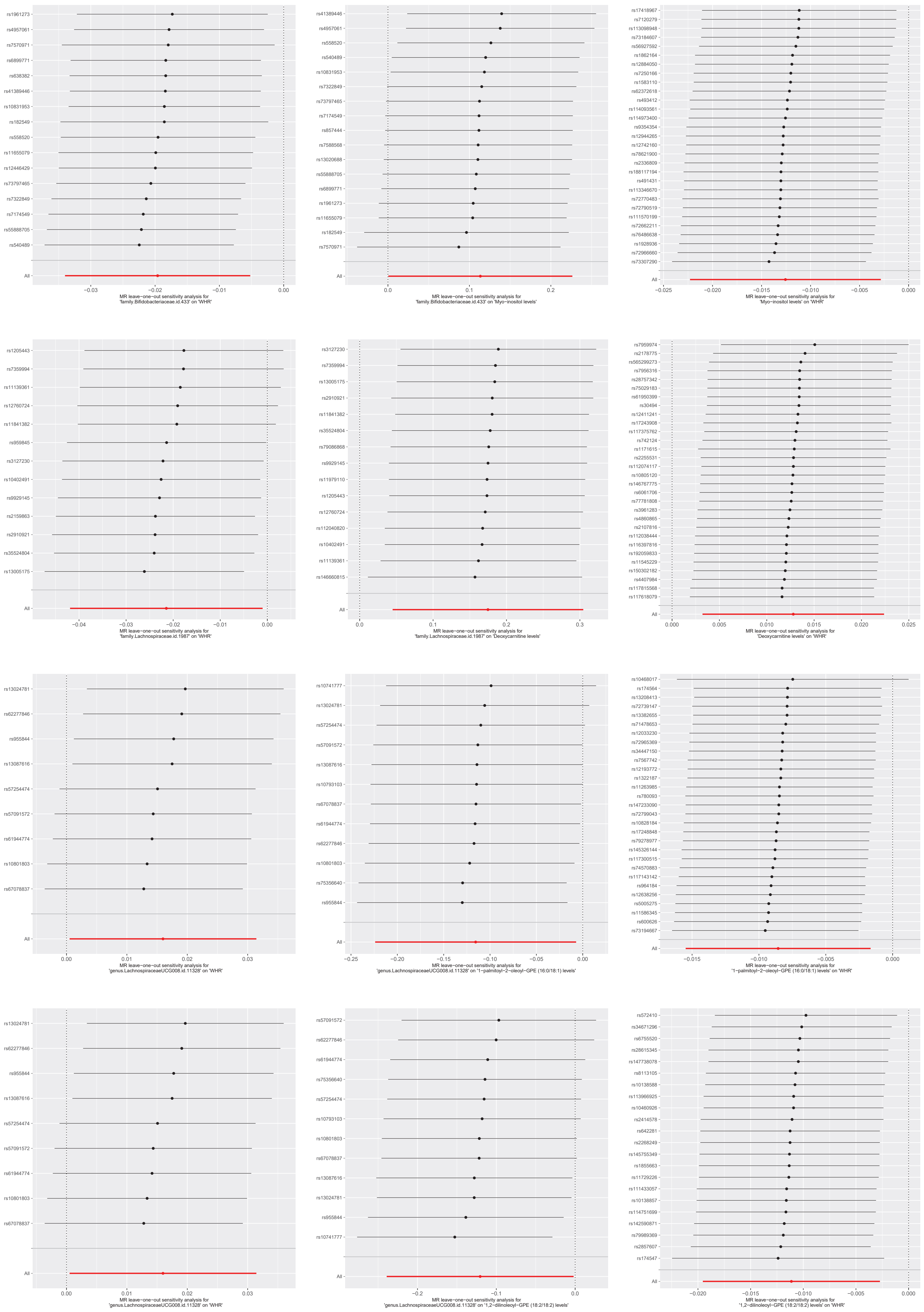

MR leave−one−out sensitivity analysis for 'family.Bifidobacteriaceae.id.433' on 'WHR'
MR leave−one−out sensitivity analysis for 'family.Bifidobacteriaceae.id.433' on 'Myo−inositol levels'
MR leave−one−out sensitivity analysis for 'Myo−inositol levels' on 'WHR'
MR leave−one−out sensitivity analysis for 'family.Lachnospiraceae.id.1987' on 'WHR'
MR leave−one−out sensitivity analysis for 'family.Lachnospiraceae.id.1987' on 'Deoxycarnitine levels'
MR leave−one−out sensitivity analysis for 'Deoxycarnitine levels' on 'WHR'
MR leave−one−out sensitivity analysis for 'genus.LachnospiraceaeUCG008.id.11328' on 'WHR'
MR leave−one−out sensitivity analysis for 'genus.LachnospiraceaeUCG008.id.11328' on '1−palmitoyl−2−oleoyl−GPE (16:0/18:1) levels'
MR leave−one−out sensitivity analysis for '1−palmitoyl−2−oleoyl−GPE (16:0/18:1) levels' on 'WHR'
MR leave−one−out sensitivity analysis for 'genus.LachnospiraceaeUCG008.id.11328' on 'WHR'
MR leave−one−out sensitivity analysis for 'genus.LachnospiraceaeUCG008.id.11328' on '1,2−dilinoleoyl−GPE (18:2/18:2) levels'
MR leave−one−out sensitivity analysis for '1,2−dilinoleoyl−GPE (18:2/18:2) levels' on 'WHR'

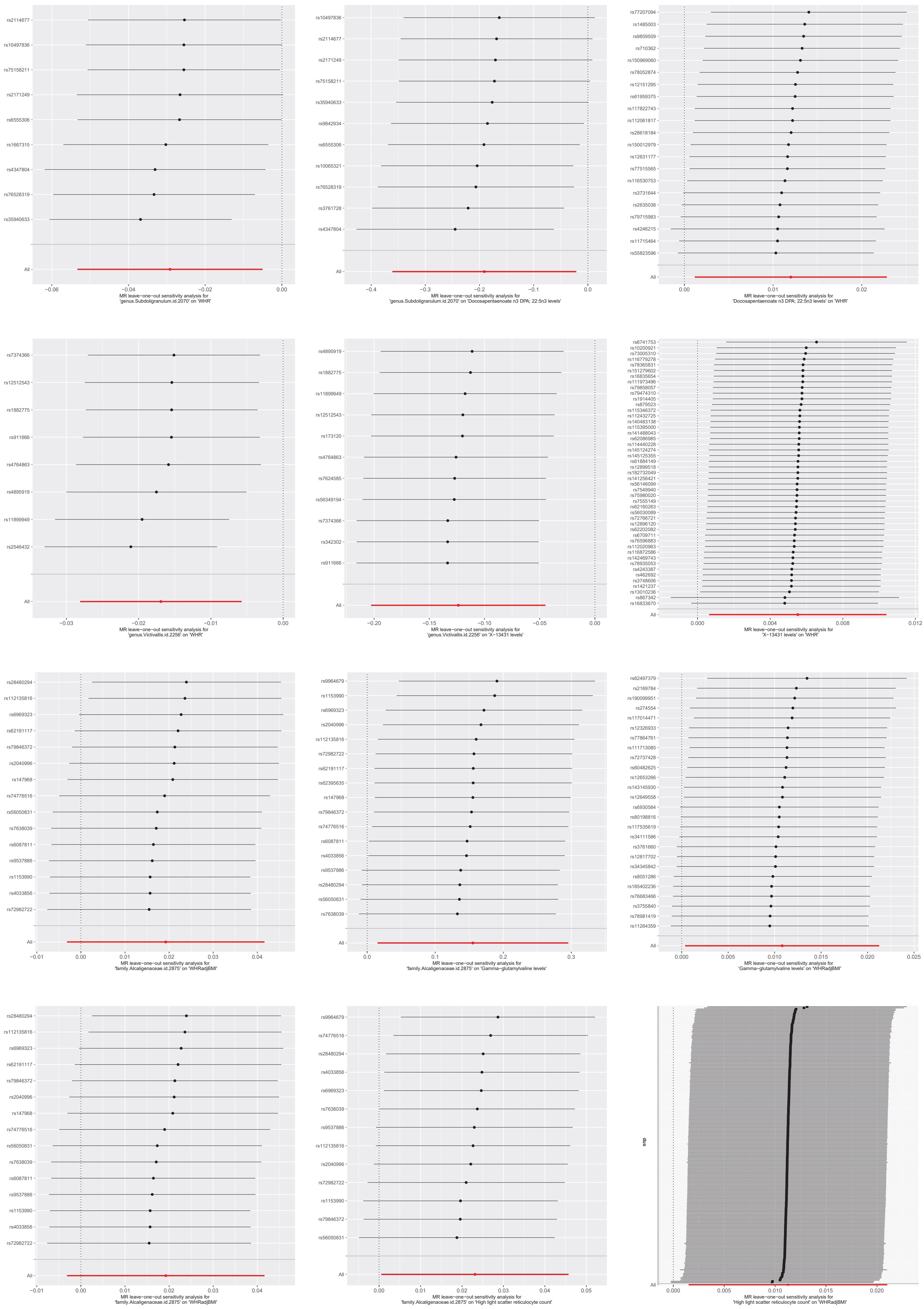
rs2114677
rs10497836
rs75158211
rs2171249
rs6555306
rs1667315
rs4347804
rs76528319
rs35940633
All
−0.06
−0.04
−0.02
0.00
MR leave−one−out sensitivity analysis for
'genus.Subdoligranulum.id.2070' on 'WHR'
rs10497836
rs2114677
rs2171249
rs75158211
rs35940633
rs9842934
rs6555306
rs10065321
rs76528319
rs3761728
rs4347804
All
−0.4
−0.3
−0.2
−0.1
0.0
MR leave−one−out sensitivity analysis for
'genus.Subdoligranulum.id.2070' on 'Docosapentaenoate n3 DPA; 22:5n3 levels'
rs77207094
rs1485003
rs9859509
rs710362
rs150969060
rs78052874
rs12151295
rs61959375
rs117822743
rs112061817
rs28618184
rs150012979
rs12631177
rs77515565
rs116530753
rs3731644
rs2635038
rs79715983
rs4246215
rs11715464
rs55823596
All
0.00
0.01
0.02
MR leave−one−out sensitivity analysis for
'Docosapentaenoate n3 DPA; 22:5n3 levels' on 'WHR'
rs7374366
rs12512543
rs1882775
rs911666
rs4764863
rs4895919
rs11899949
rs2546432
All
−0.03
−0.02
−0.01
0.00
MR leave−one−out sensitivity analysis for
'genus.Victivallis.id.2256' on 'WHR'
rs4895919
rs1882775
rs11899949
rs12512543
rs173120
rs4764863
rs7624585
rs56349194
rs7374366
rs342302
rs911666
All
−0.20
−0.15
−0.10
−0.05
0.00
MR leave−one−out sensitivity analysis for
'genus.Victivallis.id.2256' on 'X−13431 levels'
rs6741753
rs10200921
rs73005310
rs116779278
rs78365831
rs151279602
rs16835654
rs111973496
rs79858057
rs79474310
rs1914405
rs879523
rs115346372
rs112432725
rs140483138
rs115395000
rs141488043
rs62086985
rs114440228
rs145124274
rs145125355
rs61884149
rs12899518
rs182732049
rs141256421
rs56146099
rs7549940
rs75980020
rs7555149
rs62160263
rs56030089
rs72766721
rs12896120
rs62202082
rs6709711
rs76596883
rs112020963
rs116872586
rs142469743
rs78935053
rs4243387
rs462692
rs3748606
rs1421237
rs13010236
rs867342
rs16833670
All
0.000
0.004
0.008
0.012
MR leave−one−out sensitivity analysis for
'X−13431 levels' on 'WHR'
rs28480294
rs112135816
rs6969323
rs62191117
rs79846372
rs2040996
rs147968
rs74776516
rs56050831
rs7638039
rs6087811
rs9537886
rs1153990
rs4033856
rs72982722
All
−0.01
0.00
0.01
0.02
0.03
0.04
MR leave−one−out sensitivity analysis for
'family.Alcaligenaceae.id.2875' on 'WHRadjBMI'
rs9964679
rs1153990
rs6969323
rs2040996
rs112135816
rs72982722
rs62191117
rs62395635
rs147968
rs79846372
rs74776516
rs6087811
rs4033856
rs9537886
rs28480294
rs56050831
rs7638039
All
0.0
0.1
0.2
0.3
MR leave−one−out sensitivity analysis for
'family.Alcaligenaceae.id.2875' on 'Gamma−glutamylvaline levels'
rs62497379
rs2169784
rs190099951
rs274554
rs117014471
rs12326933
rs77864761
rs111713085
rs72737428
rs60482625
rs12653266
rs143145930
rs12649558
rs6930584
rs80198816
rs117535619
rs34111586
rs3761660
rs12817702
rs34345842
rs8051286
rs185402236
rs76683466
rs3755840
rs78981419
rs11264359
All
0.000
0.005
0.010
0.015
0.020
0.025
MR leave−one−out sensitivity analysis for
'Gamma−glutamylvaline levels' on 'WHRadjBMI'
rs28480294
rs112135816
rs6969323
rs62191117
rs79846372
rs2040996
rs147968
rs74776516
rs56050831
rs7638039
rs6087811
rs9537886
rs1153990
rs4033856
rs72982722
All
−0.01
0.00
0.01
0.02
0.03
0.04
MR leave−one−out sensitivity analysis for
'family.Alcaligenaceae.id.2875' on 'WHRadjBMI'
rs9964679
rs74776516
rs28480294
rs4033856
rs6969323
rs7638039
rs9537886
rs112135816
rs2040996
rs72982722
rs1153990
rs79846372
rs56050831
All
0.00
0.01
0.02
0.03
0.04
0.05
MR leave−one−out sensitivity analysis for
'family.Alcaligenaceae.id.2875' on 'High light scatter reticulocyte count'
snp
All
0.000
0.005
0.010
0.015
0.020
MR leave−one−out sensitivity analysis for
'High light scatter reticulocyte count' on 'WHRadjBMI'

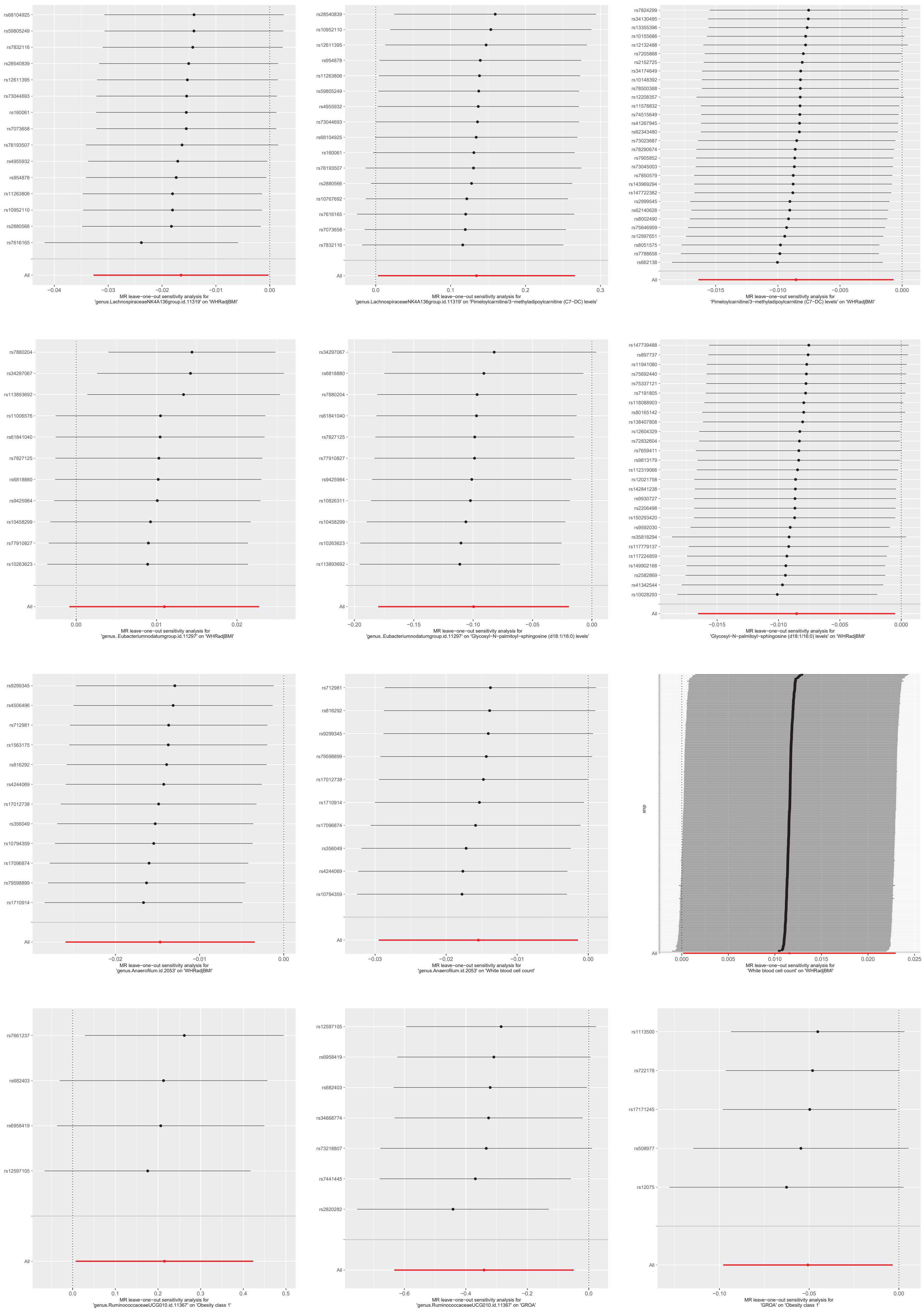
rs68104925
rs59805249
rs7832116
rs28540839
rs12611395
rs73044693
rs160061
rs7073658
rs76193507
rs4955932
rs954878
rs11263806
rs10952110
rs2880566
rs7616165
All
MR leave−one−out sensitivity analysis for
'genus.LachnospiraceaeNK4A136group.id.11319' on 'WHRadjBMI'
rs28540839
rs10952110
rs12611395
rs954878
rs11263806
rs59805249
rs4955932
rs73044693
rs68104925
rs160061
rs76193507
rs2880566
rs10767692
rs7616165
rs7073658
rs7832116
All
MR leave−one−out sensitivity analysis for
'genus.LachnospiraceaeNK4A136group.id.11319' on 'Pimeloylcarnitine/3−methyladipoylcarnitine (C7−DC) levels'
MR leave−one−out sensitivity analysis for
'Pimeloylcarnitine/3−methyladipoylcarnitine (C7−DC) levels' on 'WHRadjBMI'
rs7880204
rs34297067
rs113893692
rs11006576
rs61841040
rs7827125
rs6818880
rs9425984
rs10458299
rs77910827
rs10263623
All
MR leave−one−out sensitivity analysis for
'genus..Eubacteriumnodatumgroup.id.11297' on 'WHRadjBMI'
rs34297067
rs6818880
rs7880204
rs61841040
rs7827125
rs77910827
rs9425984
rs10826311
rs10458299
rs10263623
rs113893692
All
MR leave−one−out sensitivity analysis for
'genus..Eubacteriumnodatumgroup.id.11297' on 'Glycosyl−N−palmitoyl−sphingosine (d18:1/16:0) levels'
MR leave−one−out sensitivity analysis for
'Glycosyl−N−palmitoyl−sphingosine (d18:1/16:0) levels' on 'WHRadjBMI'
rs9299345
rs4506496
rs712981
rs1563175
rs816292
rs4244069
rs17012738
rs356049
rs10794359
rs17096874
rs79598899
rs1710914
All
MR leave−one−out sensitivity analysis for
'genus.Anaerofilum.id.2053' on 'WHRadjBMI'
rs712981
rs816292
rs9299345
rs79598899
rs17012738
rs1710914
rs17096874
rs356049
rs4244069
rs10794359
All
MR leave−one−out sensitivity analysis for
'genus.Anaerofilum.id.2053' on 'White blood cell count'
snp
All
MR leave−one−out sensitivity analysis for
'White blood cell count' on 'WHRadjBMI'
rs7661237
rs682403
rs6958419
rs12597105
All
MR leave−one−out sensitivity analysis for
'genus.RuminococcaceaeUCG010.id.11367' on 'Obesity class 1'
rs12597105
rs6958419
rs682403
rs34668774
rs73218807
rs7441445
rs2820282
All
MR leave−one−out sensitivity analysis for
'genus.RuminococcaceaeUCG010.id.11367' on 'GROA'
rs1113500
rs722178
rs17171245
rs508977
rs12075
All
MR leave−one−out sensitivity analysis for
'GROA' on 'Obesity class 1'

Figure S4 Leave-one-out plots for two-sample MR analysis of gut microbiota on obesity, gut microbiota on mediators, and mediators on obesity. The dark dots indicate effect measures from IVW MR analysis excluding the index SNPs. The red lines indicate the effect size from pooled analysis including all SNPs according to the IVW MR method.
